# GATA transcription factors drive initial Xist upregulation after fertilization through direct activation of long-range enhancers

**DOI:** 10.1038/s41556-023-01266-x

**Published:** 2023-11-06

**Authors:** Liat Ravid Lustig, Abhishek Sampath Kumar, Till Schwämmle, Ilona Dunkel, Gemma Noviello, Elodie Limberg, Raha Weigert, Guido Pacini, René Buschow, Afrah Ghauri, Maximilian Stötzel, Lars Wittler, Alexander Meissner, Edda G. Schulz

**Affiliations:** 1 Systems Epigenetics, Otto Warburg Laboratories, Max Planck Institute for Molecular Genetics, Berlin, Germany; 2 Department of Genome Regulation, Max Planck Institute for Molecular Genetics, Berlin, Germany; 3 Institute of Biotechnology, Technische Universität Berlin, Berlin, Germany; 4 Microscopy and Cryo-Electron Microscopy, Max Planck Institute for Molecular Genetics, Berlin, Germany; 5 Transgenic Unit, Max Planck Institute for Molecular Genetics, Berlin, Germany

**Keywords:** Dosage compensation, Gene regulation, Pluripotency

## Abstract

X-chromosome inactivation (XCI) balances gene expression between the sexes in female mammals. Shortly after fertilization, upregulation of Xist RNA from one X chromosome initiates XCI, leading to chromosome-wide gene silencing. XCI is maintained in all cell types, except the germ line and the pluripotent state where XCI is reversed. The mechanisms triggering Xist upregulation have remained elusive. Here we identify GATA transcription factors as potent activators of Xist. Through a pooled CRISPR activation screen in murine embryonic stem cells, we demonstrate that GATA1, as well as other GATA transcription factors can drive ectopic Xist expression. Moreover, we describe GATA-responsive regulatory elements in the *Xist* locus bound by different GATA factors. Finally, we show that GATA factors are essential for XCI induction in mouse preimplantation embryos. Deletion of GATA1/4/6 or GATA-responsive *Xist* enhancers in mouse zygotes effectively prevents Xist upregulation. We propose that the activity or complete absence of various GATA family members controls initial Xist upregulation, XCI maintenance in extra-embryonic lineages and XCI reversal in the epiblast.

## Abstract (web summary)

Through a CRISPR-activation screen, Ravid Lustig et al. show that GATA transcription factors activate long-range Xist enhancers to upregulate Xist expression and initiate X chromosome inactivation in early mouse embryos.

## Main

In female mammals, one out of two X chromosomes is silenced in a process called XCI^1^. The master regulator of XCI, the long, non-coding RNA Xist, is thus nearly ubiquitously expressed across tissues^2,3^. In mice, Xist is upregulated shortly after fertilization and expressed in all cells with the exception of the pluripotent state and the germ line^4–6^. However, the mechanism by which Xist upregulation is initially induced and then maintained remains largely unclear.

In mice, Xist is upregulated from the paternal X chromosome shortly after fertilization, but remains repressed at the maternal allele by an H3K27me3 domain deposited in oocytes^4,5,7^. This imprinted form of XCI (iXCI) is maintained in the extra-embryonic lineages, such as the trophectoderm and the primitive endoderm, but reversed in the pluripotent cells (epiblast) of the preimplantation embryo through Xist downregulation and loss of the H3K27me3 imprint^4,5,8,9^. This allows the transition from iXCI to random XCI (rXCI), where each cell will inactivate either the paternal or the maternal X chromosome. rXCI is initiated shortly after implantation and maintained in all somatic cells^4,10^. Murine embryonic stem cells (mESCs) are a cell culture model for the pluripotent cells of the preimplantation embryo and are used to study XCI, because female lines carry two active X chromosomes and initiate rXCI upon differentiation^11–15^.

Xist expression is controlled by a large genomic region, which contains a series of long non-coding RNA loci, thought to repress (*Tsix*, *Linx*) or activate (*Jpx*, *Ftx*, *Xert*) Xist transcription mostly in *cis*^16,17^. Large (210–460 kb) single-copy *Xist*-containing transgenes (*tg53*, *tg80*), encompassing ~100 kb genomic sequence upstream of the *Xist* promoter, can recapitulate post-fertilization Xist upregulation and maintenance in extra-embryonic lineages, but not rXCI in somatic tissues^18,19^. Thus, Xist appears to be controlled in part by unique regulatory elements in different cellular settings. While enhancers responsible for post-fertilization Xist upregulation from the paternal X chromosome are unknown, we recently identified the functional Xist enhancer repertoire governing rXCI^17^. The majority of the identified elements were indeed located outside the *tg53*/*tg80* transgenes.

The enhancers that control Xist at the onset of rXCI are bound by several transcription factors (TFs) associated with the post-implantation pluripotent state such as OTX2 and SMAD2/3, which probably drive Xist upregulation in that developmental context^17^. Downregulation of Xist at the pluripotent state, before the onset of rXCI, has been attributed to the repressive action of pluripotency factors, such as NANOG, REX1 (ZFP42), OCT4 (POU5F1) and PRDM14^8,20–25^. Because REX1 is already present throughout preimplantation development, XCI initiation after fertilization requires de-repression of Xist through the E3 ubiquitin ligase RNF12 (RLIM), which targets REX1 for degradation^25–27^. However, the activating mechanisms that underlie post-fertilization Xist upregulation from the paternal X chromosome remain unknown.

Here we perform a pooled CRISPR activation (CRISPRa) screen in mESCs to identify additional Xist regulators. Although the screen was initially aimed at finding rXCI regulators, the strongest hit, GATA1, led us to identifying an important mechanism driving Xist upregulation from the paternal X during iXCI. We show that all members of the GATA TF family can drive ectopic Xist upregulation in mESCs. We identify distal enhancer elements that mediate GATA-dependent Xist expression, which are bound by different GATA TFs in extra-embryonic cell lines. Finally, we demonstrate that either a simultaneous zygotic knock-out of *Gata1*, *Gata4* and *Gata6* or the deletion of two GATA-responsive long-range *Xist* enhancers largely preclude post-fertilization Xist upregulation. The joint action of different GATA TFs thus drives initial Xist upregulation after fertilization and their absence in the epiblast might contribute to X reactivation.

## Results

### Pooled CRISPR screen identifies unknown Xist regulators

**Fig. 1:**
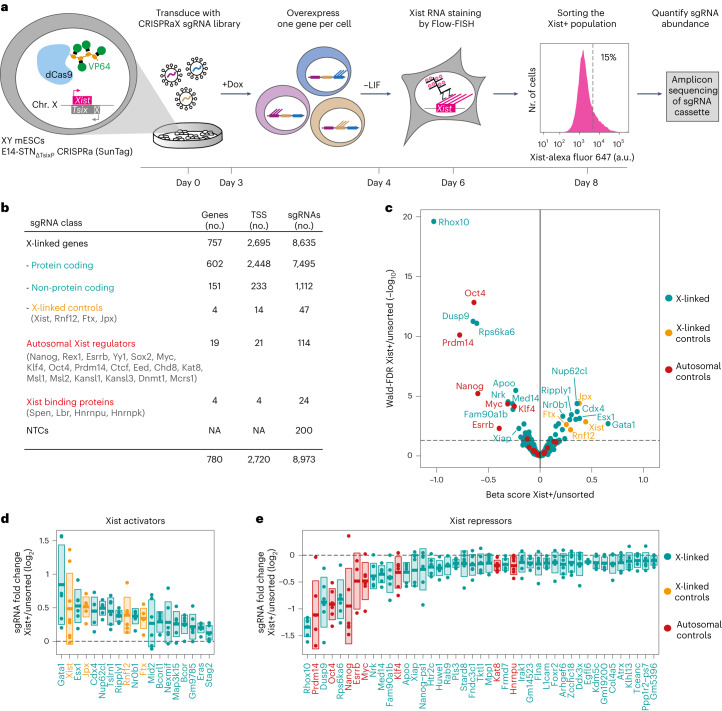
Pooled CRISPR activation screen identifies unknown Xist regulators. **a**, Schematic depiction of the CRISPRa screen workflow. A male ESC line with a deletion of the major *Tsix* promoter and a stably integrated doxycycline-inducible CRISPRa SunTag system (E14-STN_ΔTsix_) was transduced with a custom sgRNA library targeting X-chromosomal genes (CRISPRaX). Following puromycin selection, the cells were treated with doxycycline (Dox) to overexpress one gene per cell, and differentiated by LIF withdrawal (–LIF) to induce Xist upregulation. Cells were stained with Xist-specific probes by Flow-FISH and the top 15% Xist+ cells were sorted by flow cytometry. The sgRNA cassette was amplified from genomic DNA and sgRNA abundance in the unsorted and sorted populations was determined by deep sequencing. The screen was performed in three independent replicates. **b**, Composition of the CRISPRaX sgRNA library, targeting each TSS with six sgRNAs per gene. Because a subset of guides target multiple coding and non-coding transcripts, the total number of sgRNAs is smaller than the sum of sgRNAs across categories. **c**, Volcano plot of the screen results, showing the beta-score as a measure of effect size versus Wald-FDR (MAGeCK-MLE), coloured according to gene class. The dotted line denotes Wald-FDR < 0.05. **d**,**e**, Comparison of individual sgRNA abundance (dots) in the sorted fraction compared with the unsorted population for all significantly enriched (**d**) or depleted (**e**) genes in the screen (Wald-FDR < 0.05, MAGeCK-MLE). The mean of three independent replicates is shown. Genes are ordered by their beta-score, a measure for effect size (MAGeCK-MLE). The central line depicts the mean, boxes depict the standard deviation across all sgRNAs targeting the respective gene. Only the highest scoring TSS per gene is depicted. Source numerical data are available as source data. Source Data

**Extended Data Fig. 1:**
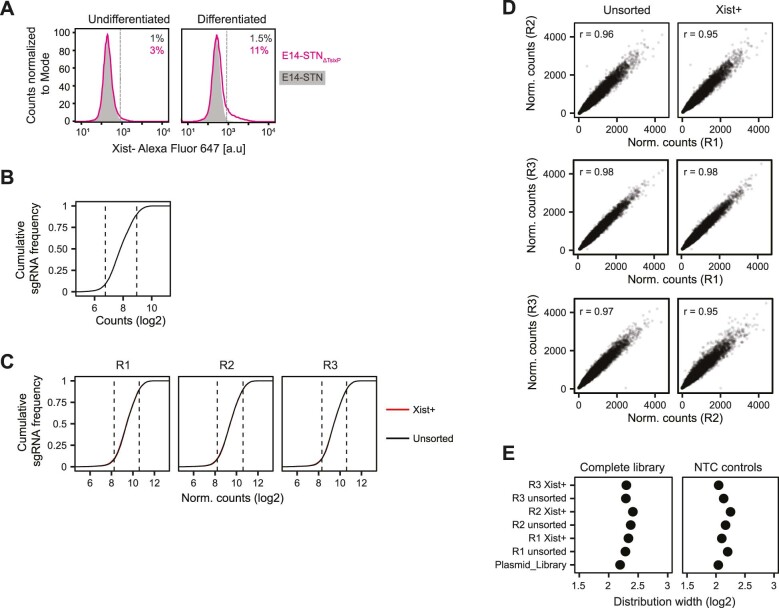
Pooled CRISPR activation screen identifies new Xist regulators. **(a)** E14-STN (grey) and E14-STN_ΔTsixP_ (pink) cells were treated with doxycycline for 3 days and were differentiated for the last 2 days by LIF withdrawal, followed by Flow-FISH with Xist-specific probes. Dashed lines mark the 99th percentile of undifferentiated E14-STN cells to separate Xist+ and Xist- cells. The percentage of Xist+ cells in each sample is indicated. **(b-c)** Cumulative frequency plot showing the distribution of sgRNA counts in the cloned sgRNA library **(b)** and in the sorted and unsorted fractions **(c)**. Dashed lines indicate the distribution width (10th and 90th percentile, quantified in **e**). **(d)** Scatterplots showing a high correlation between the replicates in the screen for each fraction as indicated. Pearson correlation coefficients between replicates are shown. **(e)** Log2 distribution width (fold change between the 10th and 90th percentiles) for all sgRNAs (left) and NTC sgRNAs only (right). The NTC distribution width was similar across samples, suggesting that sufficient library coverage was maintained during all steps of the screen. Source numerical data are available in source data. Source Data

To identify unknown Xist activators, we conducted a pooled CRISPRa screen to discover genes that, upon overexpression, induce ectopic Xist upregulation. The screen was performed in male mESCs carrying a *Tsix* promoter deletion (E14-STN_ΔTsixP_). Because Tsix is a Xist repressor, the deletion facilitates Xist upregulation, resulting in 11% of Xist-positive cells upon 2-day differentiation by withdrawal of leukemia inhibitory factor (LIF), as compared with 1.5% in the parental line (Extended Data Fig. 1a). E14-STN_ΔTsixP_ cells also carry the doxycycline-inducible SunTag CRISPRa system (Fig. 1a), which can induce strong ectopic upregulation, when recruited to a gene’s transcription start site (TSS)^28,29^. We designed and cloned a custom lentiviral sgRNA library (CRISPRaX), targeting the promoters of both protein-coding and non-coding genes on the X chromosome, as well as known Xist regulators as controls (Fig. 1b, Extended Data Fig. 1b). We focused on X-chromosomal factors since X dosage plays an important role in Xist regulation at the onset of rXCI and the screen initially was aimed at identifying rXCI regulators.

After transduction with the CRISPRaX library, resulting in genomic integration of a single sgRNA per cell, cells were differentiated for two days by LIF withdrawal. This time point was selected to reduce the likelihood of cell death caused by silencing of the single X, as both X chromosomes are still largely active at this stage, despite Xist expression already being high^30^. Cells were stained for Xist RNA using Flow-FISH and the 15% of cells with the highest signal (Xist+) were enriched via flow cytometry (Fig. 1a). Genomic DNA was isolated from the sorted and unsorted populations, and the genomically integrated sgRNA sequences were quantified by short-read sequencing (Extended Data Fig. 1c–e). Guide RNAs targeting Xist activators will be enriched in the Xist+ population, while those targeting repressors will be depleted. To identify Xist regulators, we compared sgRNA abundance in the sorted (Xist+) to the unsorted population using the MAGeCK MLE tool^31,32^ (Fig. 1c–e, Supplementary Table 1). The screen identified several known Xist activators, Xist itself (Fig. 1d, yellow) and a series of known repressors (Fig. 1e, red)^20–23,33–37^.

### GATA1 is a potent Xist activator

**Extended Data Fig. 2:**
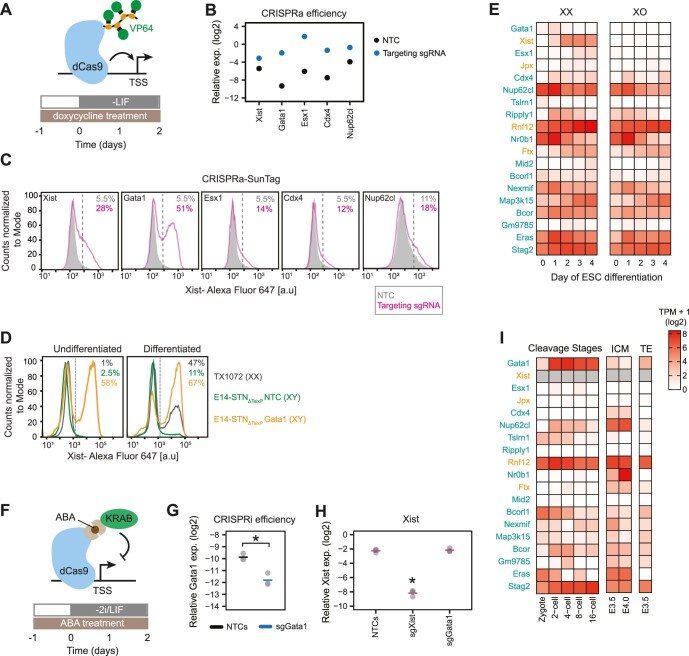
GATA1 is a potent Xist activator. **(a-c)** Individual overexpression of screen hits with CRISPRa in E14-STN_ΔTsixP_ mESCs using a single guide RNA per gene that had performed well in the screen. **(a)** The cells were treated with doxycycline 24 h before differentiation by LIF withdrawal for 2 days. **(b)** Expression levels of the targeted genes were measured by qRT-PCR. **(c)** Xist expression measured by Flow-FISH. Dashed lines mark the 99th percentile of undifferentiated NTC-transduced E14-STN_ΔTsixP_ cells (Xist- population). The percentage of Xist+ cells is indicated. **(d)** Xist expression was measured via Flow-FISH in female TX1072 cell line and in male E14-STN_ΔTsixP_ cells transduced with multiguide expression vectors of three sgRNAs against the *Gata1* promoter region or with NTCs. TX1072 cells were cultured in naive conditions (2i/LIF) and E14-STN_ΔTsixP_ in conventional ESC medium (LIF). The cells were differentiated (2i/LIF or LIF withdrawal) for 2 days. E14-STN_ΔTsixP_ were treated with doxycycline 24 h before and during differentiation. Dashed lines mark the 99th percentile of the TX1072 undifferentiated (2i/LIF) sample and the percentage of Xist+ cells in each sample is indicated. **(e)** Heatmap showing expression levels assessed by RNA-seq (mean of 3 biological replicates) of the most enriched genes in the screen (Fig. 1d) in XX and XO TX1072 mESCs differentiated by 2i/LIF withdrawal. **(f-h)** Gata1 knock-down by CRISPRi in female mESCs. **(f)** Schematic representation of an ABA-inducible CRISPRi system in female TX-SP107 mESCs. Gata1 knock-down efficiency **(g)** and effect on Xist **(h)** quantified by qRT-PCR after 2 days of differentiation. SgRNAs targeting the *Xist* TSS and NTCs were included as controls. Horizontal dashes indicate the mean of 3 biological replicates (dots); asterisks indicate p < 0.05 for two-sided paired Student’s T-test. **(i)** Expression of screen hits during preimplantation development^42,43^. Xist could not be quantified (grey) because the employed protocol was not strand-specific, such that Xist could not be distinguished from its antisense transcript Tsix. In **(e)** and **(i)** Xist and known Xist regulators are coloured in yellow. Source numerical data and exact p-values are available in source data. Source Data

Among the targeted X-linked genes, we found 15 activators, which were significantly enriched, and 35 repressors, which were depleted from the sorted fraction (Wald-FDR < 0.05, MAGeCK, Fig. 1d, e, Supplementary Table 1). The top-scoring repressors were *Rhox10*, *Dusp9*, and *Rps6ka6* (Fig. 1e). While *Rhox10* has not yet been implicated in XCI to our knowledge, *Dusp9* and *Rps6ka6* likely interfere with Xist upregulation by delaying differentiation, as they inhibit the differentiation-promoting MAPK signalling pathway^38–40^. The top candidates as putative Xist activators were the transcription regulators *Gata1*, *Cdx4*, *Esx1* and the largely uncharacterized factor *Nup62cl* (Fig. 1d). To our knowledge, none of them has been linked to Xist regulation or mESC differentiation. Only Cdx4, positioned ~150 kb downstream of *Xist*, was examined for a role in Xist regulation, but deleting its promoter had no discernible effect^41^. We validated the four top-scoring genes by individual overexpression, achieving >9-fold upregulation for all genes (Extended Data Fig. 2a,b). While all tested genes increased the number of Xist-expressing cells, Gata1 led to robust Xist upregulation in the majority of cells (Extended Data Fig. 2c). Even compared to a sgRNA targeting the *Xist* promoter directly, Gata1 induced more pronounced Xist upregulation. The Gata1-induced Xist distribution actually resembled the one seen in differentiating female mESCs (Extended Data Fig. 2d, right). Although Xist is thought to be repressed in undifferentiated mESCs, Gata1 induced efficient Xist upregulation even without differentiation (Extended Data Fig. 2d, left). These observations suggest that Gata1 is an exceptionally strong Xist activator.

We then inspected expression of the identified activators during mESC differentiation within a previously generated RNA-seq data set^30^. Among the validated screen hits, only Nup62cl was well expressed at the time when Xist was upregulated, while Gata1, Cdx4 and Esx1 showed very low expression (Extended Data Fig. 2e, Supplementary Table 2). Accordingly, knock-down of the strongest activator Gata1 in female mESCs using CRISPR interference (CRISPRi) did not affect Xist upregulation upon differentiation (Extended Data Fig. 2f–h). We therefore inspected expression of screen hits at other developmental stages, by re-analysing published scRNA-seq data^42,43^. Gata1, but not Esx1 and Cdx4, were highly expressed between the 2-cell and the 16-cell stage (Extended Data Fig. 2i), suggesting a potential role in post-fertilization Xist upregulation. While the screen was initially targeted at finding rXCI regulators, the top hit might control Xist in a different cellular context, where Xist expression is imprinted.

### All GATA TFs are strong Xist activators

**Fig. 2:**
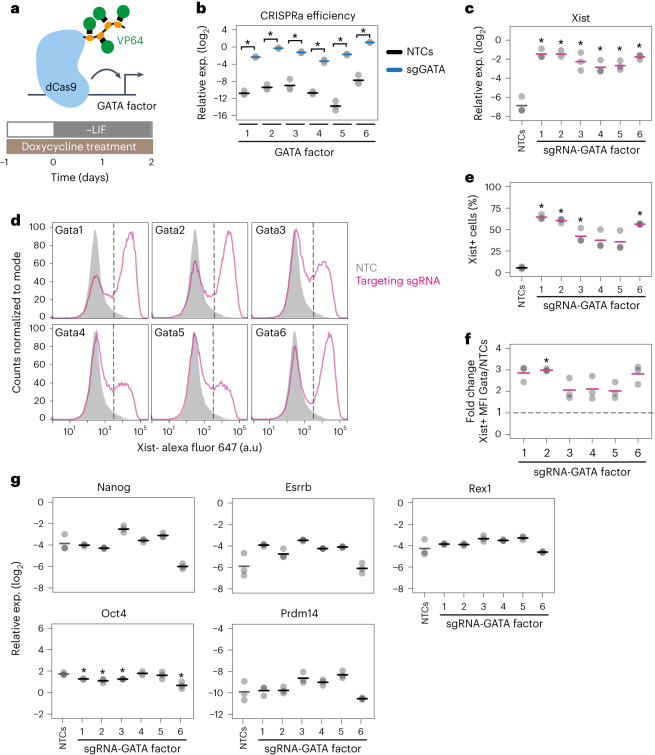
All GATA factors can induce Xist expression. **a**, Schematic representation of the cell line (E14-STN_ΔTsixP_) and experimental setup used in **b**–**g** for ectopic overexpression of GATA family members. **b**,**c**, Expression of GATA factors (**b**) and Xist (**c**) measured by qRT-PCR upon targeting each GATA TF by CRISPRa using three sgRNAs per gene. **d**–**f**, Quantification of Xist RNA by Flow-FISH, showing representative flow cytometry profiles for one replicate (**d**), the fraction of Xist-positive cells (**e**) and the mean fluorescence intensity within the Xist-positive population of the targeted GATA factors compared to the NTC (**f**) across all three replicates. In **d** the sample shaded in grey denotes cells transduced with an NTC vector. Dashed lines divide Xist+ and Xist– cells, based on the 99th percentile of undifferentiated cells, transduced with NTCs, which do not express Xist (see Extended Data Fig. 3a for gating strategy). **g**, Expression levels of pluripotency factors were assessed by qRT-PCR. In **b**, **c** and **e**–**g** the mean (horizontal dashes) of three biological replicates (dots) is shown; asterisks indicate *P* < 0.05 of a paired two-sided two-sample Student’s *t*-test for comparison to the respective NTC control (**b**, **c**, **e**, **g**) or a one-sample *t*-test (**f**) with Benjamini–Hochberg correction. Source numerical data and exact *P*-values are available as source data. Source Data

**Extended Data Fig. 3:**
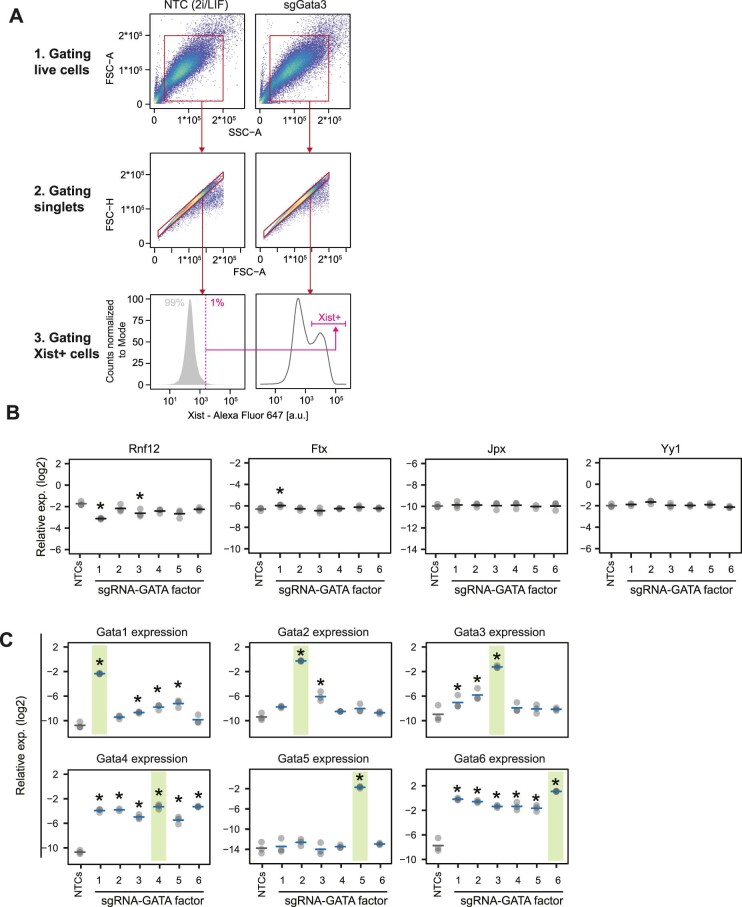
CRISPRa-mediated overexpression of GATA TFs. **(a-c)** Male E14-STN_△TsixP_ cells were transduced with multiguide expression vectors of three sgRNAs targeting the promoter of each GATA factor or with NTCs. Cells were treated with doxycycline for 3 days and differentiated for 2 days (LIF withdrawal). **(a)** The gating strategy employed for quantification of Xist by Flow-FISH. As an example, cells transduced with sgRNAs targeting Gata3 are shown (right). Undifferentiated cells (+LIF) transduced with a NTC vector (left), which do not express Xist, were used to set the gate to identify Xist+ cells (99th percentile). This gating strategy was applied in Fig. 2d–f, Extended Data Fig. 1a and Extended Data Fig. 2c, d. Steps 1 and 2 were applied in an identical manner in Fig. 1a and Fig. 4e, f. **(b**-**c)** Expression levels of known Xist regulators **(b)** and of GATA factors **(c)** were assessed by qRT-PCR. Mean (horizontal dashes) of 3 biological replicates (dots) is shown; asterisks indicate p < 0.05 of a two-sided paired Student’s T-test with Benjamini–Hochberg correction for comparison to the respective NTC control. Green areas in **(c)** indicate the GATA factor that was targeted by CRISPRa. Source numerical data and exact p-values are available in source data. Source Data

As GATA1 is part of a TF family with six members, which recognize similar DNA sequences^44^, we tested whether other family members could similarly induce Xist expression. We therefore overexpressed all six GATA factors in male mESCs using CRISPRa (Fig. 2a), and measured their effect on Xist upregulation during differentiation. Each GATA factor could be overexpressed >150-fold, resulting in 35–65% Xist+ cells and 15- to 40-fold increase in Xist RNA levels (Fig. 2b–f, Extended Data Fig. 3a). Because some GATA factors have been shown to induce differentiation in mESCs^45,46^, we tested whether they might indirectly activate Xist by reducing pluripotency factor expression. We therefore assessed how GATA overexpression affected *Nanog*, *Oct4*, *Rex1*, *Esrrb* and *Prdm14* mRNA levels, but could not detect a consistent effect (Fig. 2g). GATA-mediated Xist induction can thus not be attributed to GATA-induced differentiation. We also tested whether ectopic Xist upregulation upon GATA overexpression might be mediated by known Xist activators, but found no consistent effect on *Rnf12*, *Jpx*, *Ftx* or *Yy1*^35–37,47^ (Extended Data Fig. 3b). Because all GATA factors had a similar effect on Xist, we also analysed whether they induced each other. We indeed observed extensive cross-activation, where in particular *Gata4* and *Gata6* were induced by all other GATA factors (Extended Data Fig. 3c). Taken together, our results reveal that all 6 members of the GATA TF family are strong Xist activators, at least some of which might control Xist in a direct manner through activating the promoter or enhancer elements.

### GATA6 directly activates Xist in a dose-dependent manner

**Fig. 3:**
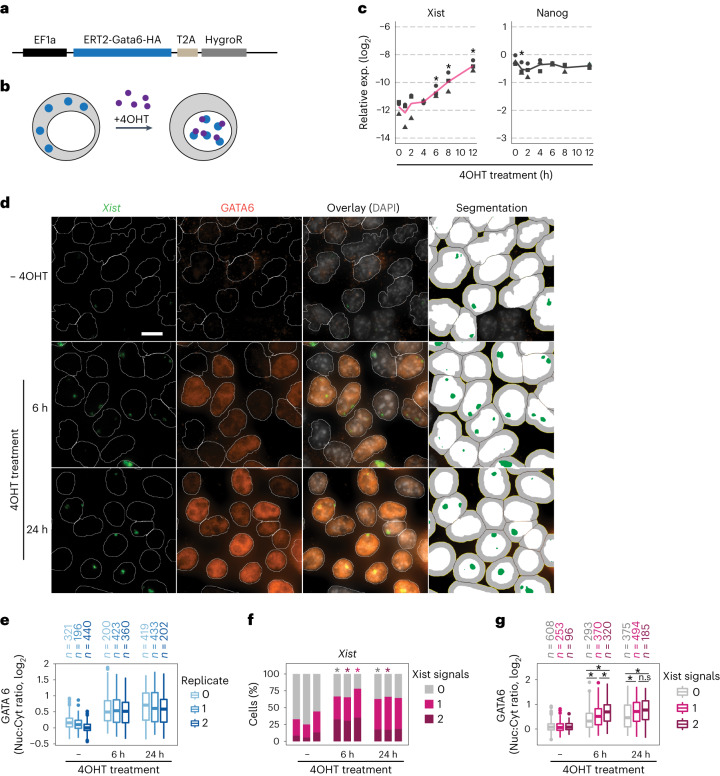
Xist is rapidly induced by GATA6 in a dose-dependent manner. **a**,**b**, Schematic representation of the ERT2-GATA6 inducible system used in **c**–**g**. Female TX-SP107 mESCs were transduced with a lentiviral vector expressing Gata6 cDNA N-terminally fused to the ERT2 domain and C-terminally tagged with HA under control of an EF1a promoter. **b**, Upon 4OHT treatment (purple), ERT2-GATA6-HA protein (blue) translocates into the nucleus. **c**, Time course of Xist and Nanog expression, assessed by qRT-PCR, upon 4OHT treatment of TX-SP107 ERT2-Gata6-HA cells, cultured in 2i/LIF medium. The black line indicates the mean of three biological replicates (symbols); asterisks indicate *P* < 0.05 using a two-sided paired Student’s *t*-test, comparing levels to the untreated control (0 h). **d**–**g**, TX-SP107 ERT2-Gata6-HA cells were grown on glass coverslips in conventional ESC medium (LIF only) for 48 h and treated with 4OHT for 6 or 24 h, followed by immunofluorescence staining (anti-HA to detect GATA6) combined with RNA-FISH (to detect Xist). 2i removal was required for the cells to flatten out to allow automated image analysis, but led to partial Xist de-repression, such that 25–44% of cells already expressed Xist without 4OHT treatment, which was significantly increased upon 4OHT treatment (**f**). Nuclei (**d**, white) and Xist signals (**d**, green) were detected by automated image segmentation and GATA6-HA staining was quantified in the nucleus and in a 2.64 μm ring around the nucleus as a proxy for the cytoplasm (right column, grey) to quantify nuclear translocation in **e** and **g**. In **e** and **f**, three biological replicates are shown, which were merged for the analysis in **g** with excluding nuclei where more than two Xist signals were detected due to segmentation errors (<10% cells). The central mark indicates the median, and the bottom and top edges of the box indicate the first and third quartiles, respectively. The top and bottom whiskers extend the boxes to a maximum of 1.5 times the interquartile range; cell numbers are indicated on top. In **f**, asterisks indicate *P* < 0.05 using a two-sided paired Student’s *t*-test; in **g** they indicate *P* < 0.01, Wilcoxon rank-sum test. Scale bar represents 10 µm. Source numerical data and exact *P*-values are available as source data. Source Data

**Extended Data Fig. 4:**
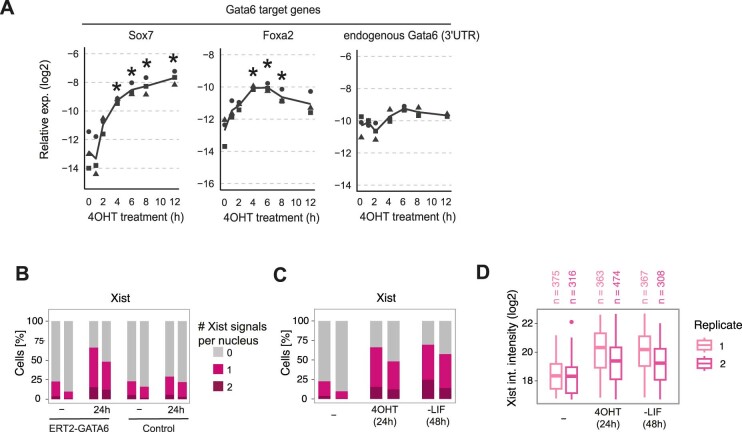
Xist is rapidly induced by GATA6 in a dose-dependent manner. **(a)** Time course of 4OHT treatment of TX-SP107 ERT2*-*Gata6-HA cells, cultured in 2i/LIF medium. Expression levels of known GATA6 target genes were measured by qRT-PCR. The black line indicates the mean of 3 biological replicates (symbols); asterisks indicate p < 0.05 using a two-sided paired Student’s T-test, comparing levels to the untreated control (0 h). **(b)** TX-SP107 ERT2-Gata6-HA cells and the parental TX-SP107 line were treated with 4OHT as described in main Fig. 3, showing that only ERT2-Gata6-HA expressing cells upregulate Xist upon 4OHT treatment. **(c, d)** ERT2-Gata6-HA cells were treated with 4OHT for 24 h as described in main Fig. 3 or were differentiated for 48 h by 2i/LIF withdrawal. The summed fluorescence intensity within the Xist cloud signals is shown in **(d)**. Both treatments induce a comparable frequency of Xist expression **(c)** and signal strength **(d)**. In **(d)** the central mark indicates the median, and the bottom and top edges of the box indicate the first and third quartiles, respectively. The top and bottom whiskers extend the boxes to a maximum of 1.5 times the interquartile range; cell numbers are indicated on top. In **(b**-**d)** 2 biological replicates are shown with excluding nuclei with >2 Xist signals due to segmentation errors (<10% of nuclei). Source numerical data are available in source data. Source Data

To test whether a GATA factor could indeed directly induce Xist expression, we established a system that allowed rapid activation of a GATA TF to then follow the dynamics of Xist upregulation. We chose GATA6, because it is an important regulator of the primitive endoderm lineage, where iXCI is maintained^48^. We generated a female mESC line stably expressing hemagglutinin (HA)-tagged Gata6 cDNA N-terminally fused to the tamoxifen-inducible oestrogen receptor (ERT2) domain (Fig. 3a). ERT2-GATA6 is retained in the cytoplasm and translocates into the nucleus upon treatment with 4-hydroxytamoxifen (4OHT; Fig. 3b). The cells were cultured in 2i/LIF conditions, where Xist is repressed, and treated with 4OHT for 12 h. From 6 h onwards, Xist levels significantly increased, with no impact on the pluripotency factor Nanog (Fig. 3c). We also assessed expression of three putative direct GATA6 target genes^49^, two of which were significantly upregulated after 4 h of 4OHT treatment (*Sox7* and *Foxa2*, Extended Data Fig. 4a). The fact that upregulation of these genes only slightly precedes Xist upregulation, further supports the idea that GATA6 can directly induce Xist. We cannot, however, exclude that other GATA6 target genes might additionally reinforce Xist upregulation.

To further characterize GATA6-dependent Xist regulation, we analysed the relationship between nuclear GATA6 and Xist expression on the single-cell level. We performed immunofluorescence staining of HA-tagged ERT2-GATA6 combined with RNA-fluorescence in situ hybridization (RNA-FISH) for Xist (IF-FISH) after 6 h and 24 h of 4OHT treatment (Fig. 3d). Through automated image segmentation, we quantified GATA6 staining within and around the nucleus to estimate nuclear and cytoplasmic GATA6 levels (Fig. 3d). Nuclei were segmented using DNA staining, and a ~2.5 μm ring was drawn around each nucleus, with reduced width for close nuclei. This ring served as an approximation for the cytoplasm, enabling us to calculate the ratio between nuclear and cytoplasmic signals (referred to as the nuc:cyt ratio) as an indicator of GATA6 nuclear accumulation. Although GATA6 expression levels appeared variable across cells, the nuc:cyt ratio was clearly increased in the majority of cells after 6 h of 4OHT treatment (Fig. 3e), accompanied by an increase in Xist-expressing cells (Fig. 3f), which was not observed in the parental line without ERT2-GATA6 expression (Extended Data Fig. 4b). When analysing the relationship between GATA6 levels and the Xist pattern, we observed that higher GATA6 nuc:cyt ratios correlated with more Xist signals, indicating that GATA6 induces Xist in a dosage-dependent manner (Fig. 3g). Moreover, analysis of the signal intensity revealed that the GATA6-induced expression level at 24 h was comparable to the peak levels observed in female mESCs after 48 h of differentiation (Extended Data Fig. 4c,d). The observed potent and dosage-dependent Xist upregulation further supports GATA6 as a direct Xist activator.

### GATA6 regulates Xist through a distal enhancer element

**Fig. 4:**
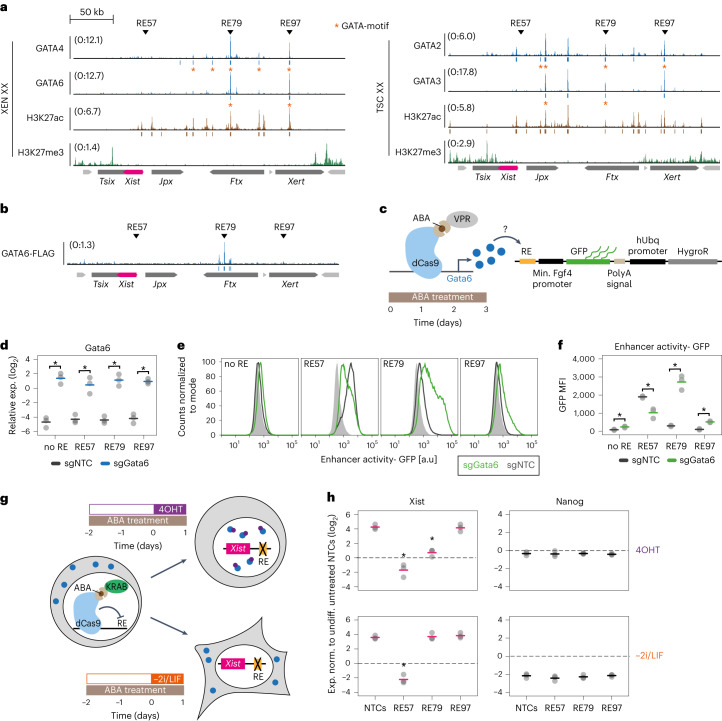
GATA6 regulates Xist by binding to a distal enhancer element. **a**, Histone modifications and binding profiles for selected GATA TFs in female XEN (left) and TS cells (right), profiled by CUT&Tag. Peaks containing the respective GATA factor binding motif (*P* < 0.001, FIMO) are marked with an orange asterisk. Two or three biological replicates were merged. **b**, Published ChIP-seq data in mESCs overexpressing GATA6^49^. Arrowheads in **a** and **b**, denote two regulatory elements (RE), RE79 and RE97, which are bound by all four tested GATA factors and the promoter-proximal RE57, which is not bound by GATA factors. Significant peaks (*q* < 0.05, MACS2) are indicated below the tracks. **c**–**f**, Effect of GATA6 overexpression on a GFP reporter under control of different REs. TX-SP106 mESCs carrying a stably integrated ABA-inducible CRISPRa (VPR) system (**c**), were cultured in conventional ESC conditions and transduced with multiguide expression vectors of three sgRNAs against Gata6 or with NTCs. Cells were transduced with either the empty or RE-containing (RE57, RE79 and RE97) lentiviral FIREWACh enhancer–reporter vector and treated with ABA for 3 days (**c**). Upregulation of Gata6 was measured by qRT-PCR (**d**) and GFP levels were assessed by flow cytometry (**e** and **f**). In **e**, light grey represents the cells’ autofluorescence. **g**,**h**, Repression of REs through an ABA-inducible CRISPRi system and simultaneous GATA6 overexpression. Female TX-SP107 ERT2-Gata6-HA mESCs were cultured in 2i/LIF conditions and transduced with multiguide expression vectors of three or four sgRNAs against REs or with NTCs. The cells were treated for 3 days with ABA to repress the respective RE and one day before harvesting, the cells were either differentiated (bottom, –2i/LIF, GATA6-independent Xist upregulation) or treated with 4OHT (top, GATA6-dependent Xist upregulation). Xist and Nanog mRNA levels were assessed by qRT-PCR. Samples were normalized to undifferentiated NTC controls not treated with 4OHT. In **d**, **f** and **h** horizontal dashes indicate the mean of three biological replicates (dots); asterisks indicate *P* < 0.05 using a two-sided paired Student’s *t*-test for comparison to the respective NTC sample. The exact *P*-values are 0.009, 0.02, 0.007 and 0.008 (**d**); 0.03, 0.02, 0.009 and 0.006 (**f**); 0.003, 0.002 and 0.5 (**h**, Xist, 4OHT); 0.001, 0.5 and 0.05 (**h**, Xist, –2i/LIF). Source numerical data are available as source data. Source Data

**Extended Data Fig. 5:**
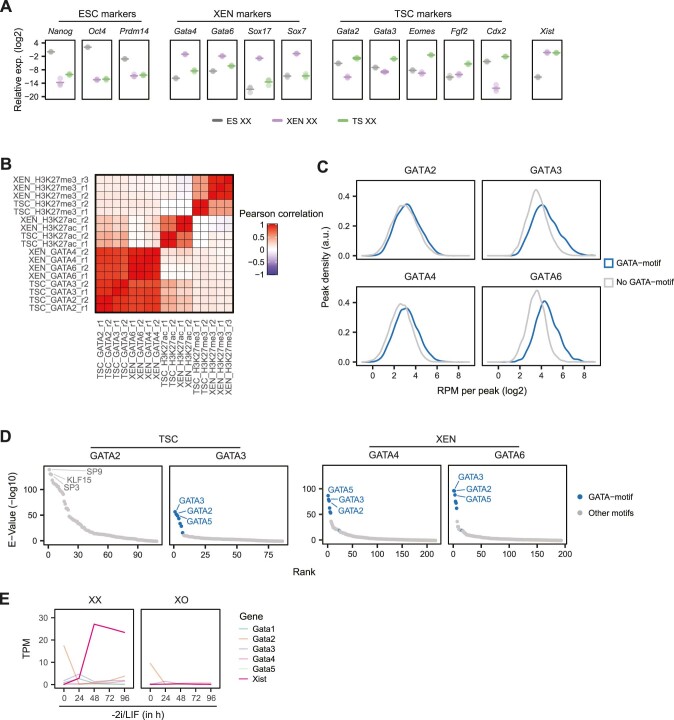
GATA factor profiling by CUT&Tag in XEN and TSCs and by RNA-seq in mESCs. **(a)** Relative expression levels of various marker genes of ESCs, XEN and TS cells as indicated and of Xist, measured via qRT-PCR in female TX1072 ESCs, XEN and TS cells. Mean (dash) of 3 biological replicates (dots) is shown. **(b)** Pearson correlation coefficient between all CUT&Tag samples. The heatmap is ordered according to hierarchical clustering of the correlations. Correlation between biological replicates was high and the samples showed the expected correlation patterns. **(c)** Density of RPM values per peak in each condition of the GATA CUT&Tag data. The data is split in peaks containing (blue) or not containing (grey) the respective GATA-motif (p < 0.001, FIMO). While peaks with a motif were clearly stronger for GATA6 and GATA3, and to a slightly lesser extent also for GATA4, no difference was observed for GATA2. **(d)** Enrichment of TF-binding motifs within peaks identified for the different GATA TFs using AME. Binding motifs were ranked according to their E-values, a measure of the statistical enrichment of the respective motif. All binding motifs with an -log10(E-value) < 10 are shown. All GATA-family binding motifs are coloured in blue. Additionally, the 3 most enriched motifs per sample are labelled. For GATA3, GATA4 and GATA6 all top-ranking motifs were members of the GATA family, while no GATA motifs were found for GATA2. These analyses suggest that GATA3, GATA4 and GATA6 can be profiled reliably by CUT&Tag, while the data for GATA2 should be interpreted with caution. **(e)** Expression pattern of GATA TFs and Xist in differentiating mESCs (2i/LIF-withdrawal) with one (TX1072 XO H7/A3) or two X-chromosomes (TX1072 XX) measured by RNA-seq. Source numerical data are available in source data. Source Data

Next, we aimed at identifying regulatory elements within *Xist’s* cis-regulatory landscape that mediate GATA-dependent regulation. As a first step, we identified binding sites for GATA factors in female extra-embryonic cell lines, which express different sets of GATA TFs and maintain Xist expression in an imprinted manner^11,12,50^. We analysed GATA2 and GATA3 in a trophoblast stem (TS) cell line and GATA4 and GATA6 in an extra-embryonic endoderm stem (XEN) cell line through CUT&Tag^51^. We also profiled the repressive histone modification H3K27me3, which constitutes the Xist imprint^7,52^, and the H3K27ac mark as a proxy for active enhancers (Fig. 4a, Extended Data Fig. 5a–d, Supplementary Table 3).

In both cell types we detected a series of H3K27ac peaks in a ~200 kb region upstream of the *Xist* promoter, which was largely devoid of H3K27me3. Notably, this region is covered by the maternal H3K27me3 imprint up to the blastocyst stage^7^, further supporting the presence of *Xist* enhancers in that region. The maternal H3K27me3 domain however, appears to be lost in TS and XEN cells, in agreement with a previous study in TS cells^53^. For the collected GATA binding profiles we performed a series of quality controls (Extended Data Fig. 5b–d, Methods). With the exception of GATA2, CUT&Tag appeared to primarily detect the expected binding sites. For GATA6 we observed two prominent binding sites in the 200 kb region upstream of *Xist*, both of which overlapped with H3K27ac peaks (Fig. 4a). Both regions also appeared to be bound by GATA2 and GATA3 in TS cells and by GATA4 in XEN cells (Fig. 4a). These binding sites correspond to regulatory elements (RE) 79 and 97, which we have previously tested for *Xist* enhancer activity in differentiating mESCs through a pooled CRISPRi screen^17^. RE97, but not RE79 was identified as a functional enhancer during the onset of rXCI in that screen. In a published GATA6 ChIP-seq data set^49^, upon 36 h GATA6 overexpression in mESCs, RE79 but not RE97 was strongly bound (Fig. 4b). The GATA binding pattern thus seems to be more restricted in mESCs compared to extra-embryonic cell lines.

To investigate whether GATA6 can indeed activate RE79 and potentially RE97, we tested whether GATA6 overexpression could induce a GFP reporter controlled by these potential enhancer elements (Fig. 4c–f). As a negative control, we also included RE57, which is located proximal to the *Xist* promoter and plays an important role in Xist regulation^17,54^, but is not bound by GATA TFs (Fig. 4a). We cloned the three genomic regions (600–900 bp) into a lentiviral enhancer–reporter plasmid, which was then co-expressed with a CRISPRa system to allow ectopic GATA6 upregulation^55,56^ (Fig. 4c). RE79 and RE97 showed low reporter activity in NTC (non-targeting control)-transduced ESCs, whereas RE57 exhibited high basal activity (Fig. 4e, black). A greater than 30-fold overexpression of *Gata6* mRNA (Fig. 4d) resulted in a strong 9- and 5-fold increase for RE79 and RE97, respectively (Fig. 4e, f), showing that these genomic loci constitute indeed GATA6-dependent enhancer elements. For RE57 no increase in GFP levels upon GATA6 overexpression was detected, instead we observed a decrease (Fig. 4e,f), potentially due to indirect effects by modulation of the cellular differentiation state.

To test the functional importance of RE79 and RE97 in their endogenous genomic context, we next aimed to block their activation by CRISPRi and then probe the effect on GATA6-dependent Xist upregulation. We again made use of our female ERT2-GATA6 transgenic mESC line (Fig. 3) and co-expressed our CRISPRi system. Through simultaneous expression of three or four sgRNAs targeting one RE we blocked activation of RE79 and RE97 as well as the promoter-proximal RE57 as a control. Two days later, the cells were either treated with 4OHT to induce GATA6 translocation or differentiated to induce Xist upregulation in a GATA6-independent manner (Fig. 4g). Both, GATA6 induction (+4OHT) as well as differentiation (–2i/LIF) led to ~20-fold Xist upregulation in NTC-transduced control cells after 24 h (Fig. 4h). While targeting RE57 completely blocked Xist upregulation under both conditions, RE79 abolished GATA6-dependent Xist upregulation nearly completely (Fig. 4h, top), but did not affect differentiation-induced Xist expression, when GATA6 remained in the cytoplasm (Fig. 4h, bottom). By contrast, targeting RE97 had no detectable effect in either context, suggesting that although RE97 can be bound and regulated by GATA factors in other cell types, it does not regulate Xist via this mechanism in mESCs. The observation that RE97 also did not affect Xist expression upon 1 day of differentiation is in agreement with our previous finding that Xist is only affected by a deletion of the RE97-containing region from day 2 of differentiation onwards^17^. These results suggest that GATA6 induces Xist expression primarily through RE79, when over-expressed in ESCs, in agreement with its binding pattern in that cell line (Fig. 4b). The GATA/RE79-dependent mode of regulation appears to be sufficient, but not necessary for Xist upregulation, as GATA TFs are absent during early mESC differentiation (Extended Data Fig. 5e) and RE97 is dispensable. In other cellular contexts, where GATA TFs are endogenously expressed, additional GATA binding sites might mediate Xist regulation.

### GATA factors upregulate Xist after fertilization in vivo

**Fig. 5:**
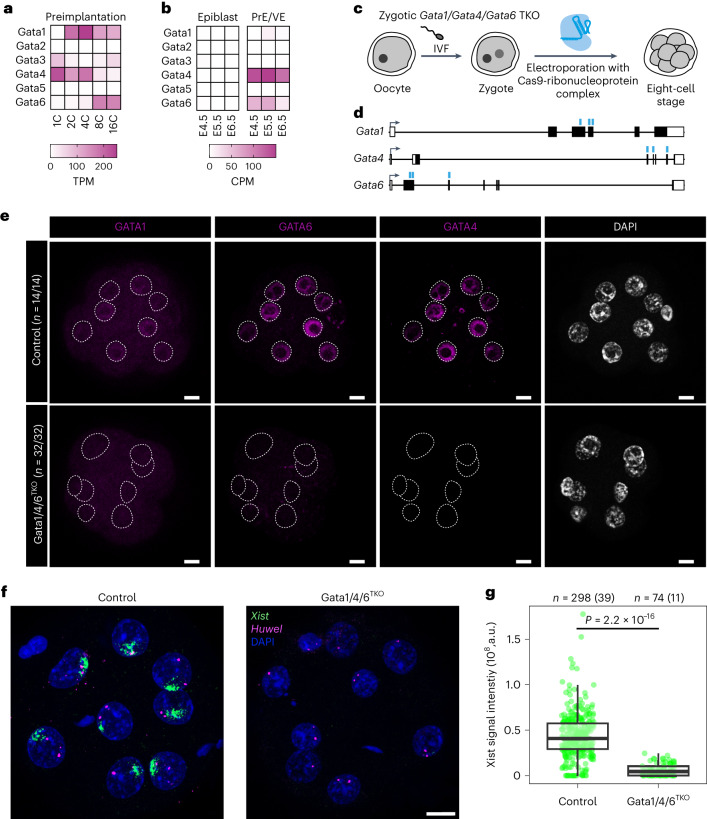
GATA factors are required for initial Xist upregulation *in vivo*. **a**,**b**, Expression of GATA TFs during early development assessed by scRNA-seq^42,57^. C, cell; PrE, primitive endoderm; VE, visceral endoderm. **c**–**g**, Zygotic TKO of Gata1, Gata4 and Gata6. **c**, Schematic depiction of the experimental workflow, where zygotes, generated by IVF were electroporated with Alt-R CRISPR/Cas9 ribonucleoprotein complex pre-assembled with three crRNAs targeting the Gata1, Gata4 and Gata6 coding sequences. Embryos were allowed to develop to the eight-cell stage. **d**, Schematic depiction of Gata1, Gata4 and Gata6 genomic loci with regions targeted by crRNAs shown as blue lines. **e**, Staining of the indicated GATA TFs. Dashed lines represent the nuclei as detected by DAPI staining. For the numbers indicated, two biological replicates were merged. **f**,**g**, RNA-FISH for Xist and the X-linked Huwe1 gene (nascent transcript) at the eight-cell stage. Only female embryos (two Huwe1 signals) were included in the analysis. In **g**, the summed fluorescence intensity within the automatically detected Xist clouds is shown for individual cells. Embryos from two biological replicates were pooled (individual replicates are shown in Extended Data Fig. 7b). Statistical comparison was performed with a two-sided Wilcoxon ranksum test. The central mark indicates the median, and the bottom and top edges of the box indicate the first and third quartiles, respectively. The top and bottom whiskers extend the boxes to a maximum of 1.5 times the interquartile range; cell (embryo) numbers are indicated on top. The scale bars in **e** and **f** represent 10 μm. Source numerical data are available as source data. Source Data

**Extended Data Fig. 6:**
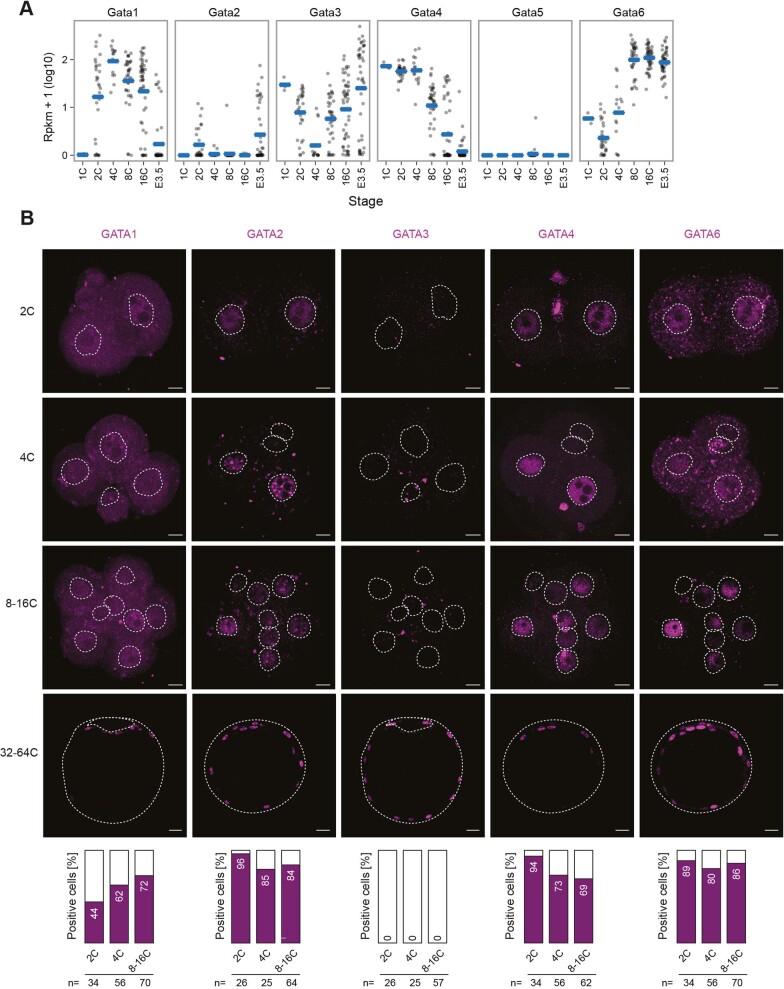
Multiple GATA TFs are expressed during mouse preimplantation development. **(a)** Expression of GATA TFs assessed by scRNA-seq across different stages of early mouse development^42^. Horizontal dashes indicate the mean of 24 (1C), 180 (2C), 84 (4C), 222 (8C), 300 (16C) and 258 (E3.5) cells. **(b)** Protein staining of all GATA TFs except GATA5 in preimplantation mouse embryos (stages indicated). Nuclei were detected by DAPI staining and their contour is marked (dashed line). Bar plots show the percentages of positive nuclei for the respective GATA protein. Percentages represent the mean of two biological replicates. The number of nuclei counted is shown below the plots. Scale bars represent 10 μm, scale bars for 32-64 C are 20 μm. Source numerical data are available in source data. Source Data

Having demonstrated the potency of GATA factors as Xist activators, we examined the physiological significance of GATA-dependent Xist regulation. To this end, we first analysed GATA expression patterns during early development at the level of transcripts and proteins through re-analysis of published single-cell RNA-seq data^42,57^ and immunofluorescence staining (Fig. 5a,b, Extended Data Fig. 6). In agreement with previous reports, multiple GATA factors were expressed at all stages of preimplantation development with the exception of the pluripotent epiblast^48^. The observed expression profile aligns precisely with the documented pattern of Xist expression in early embryos. Xist is known to be upregulated shortly after fertilization and is downregulated only in pluripotent cells^4,5,9^.

**Extended Data Fig. 7:**
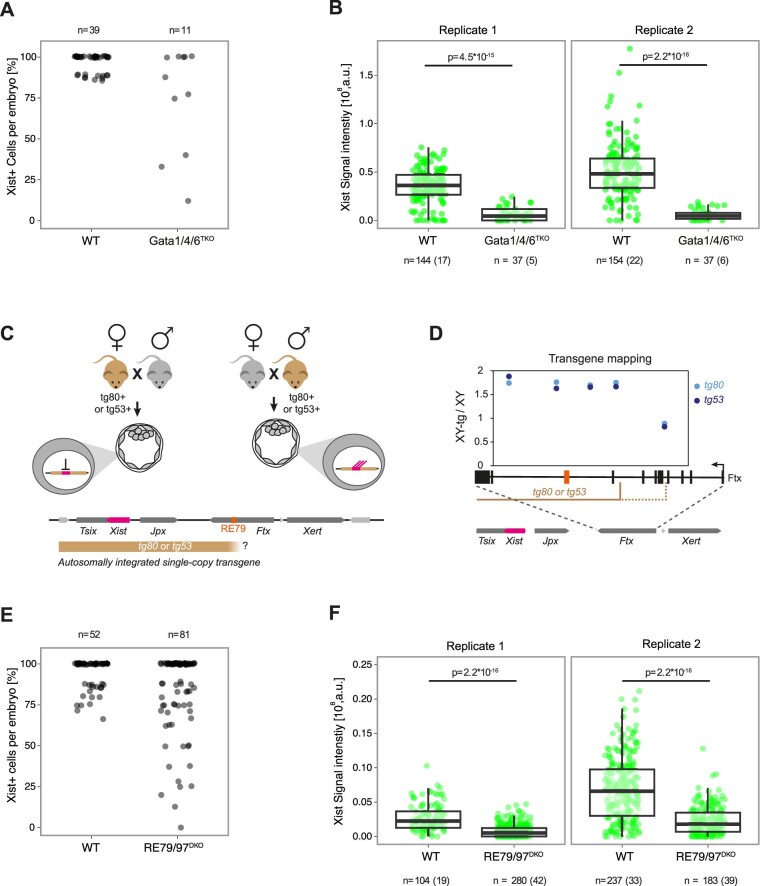
The role of GATA factors *in vivo*. **(a, b)** Zygotic triple knock-out (TKO) of *Gata1, Gata4* and *Gata6* as shown in main Fig. 5f. **(a)** The percentage of cells in each embryo with an Xist signal is shown at the eight-cell stage. Two biological replicates were merged. The efficiency of Xist upregulation is reduced in TKO embryos. **(b)** The summed fluorescence intensity within the automatically detected Xist clouds is shown for individual cells. Statistical comparison was performed with a two-sided Wilcoxon ranksum test. The number of cells (embryos) included in the analysis is indicated below. **(c)** The *tg80/tg53* transgenes (beige), which contain the *Xist* gene and ~100 kb of upstream genomic sequence (bottom), can reproduce imprinted Xist expression, when autosomally integrated as a single copy, as they are expressed upon paternal (right), but not upon maternal (left) transmission^18,19^. **(d)** Mapping of the telomeric end of *tg80/tg53* by qPCR on genomic DNA from XY-tg80/tg53 ESCs with primer pairs detecting different positions around RE79, as indicated below the plot. Mapping confirms that *tg80* and *tg53* contain the RE79 region. Results are expressed as relative DNA quantity with respect to XY cells without the transgene (E14-STN_ΔTsixP_). **(e, f)** Zygotic double knock-out (DKO) of RE79 and RE97 as shown in main Fig. 6c. **(e)** The percentage of cells in each embryo with an Xist signal is shown at the eight-cell stage. Two biological replicates were merged. The efficiency of Xist upregulation is reduced in DKO embryos. **(f)** The summed fluorescence intensity within the automatically detected Xist cloud is shown for individual cells. Statistical comparison was performed with a two-sided Wilcoxon ranksum test. The number of cells (embryos) included in the analysis is indicated below. In **(b)** and **(f)** the central mark indicates the median, and the bottom and top edges of the box indicate the first and third quartiles, respectively. The top and bottom whiskers extend the boxes to a maximum of 1.5 times the interquartile range. Source numerical data are available in source data. Source Data

To test whether GATA factors play a functional role in Xist regulation in early embryos, we deleted selected GATA TFs through zygotic electroporation of a Cas9 ribonucleoprotein complex. We generated triple knock-out embryos of Gata1, Gata4 and Gata6 (Gata1/4/6^TKO^), as these factors exhibited high expression levels during the first days of development (Fig. 5a–d). When assaying for GATA1/4/6 protein expression at the eight-cell stage, we found that the knock-out (KO) strategy was highly efficient. All 32 Gata1/4/6^TKO^ embryos analysed were deficient for all three factors, which were robustly detected in embryos electroporated with a control sgRNA targeting GFP (Fig. 5e). We therefore assayed Xist expression by RNA-FISH also at the eight-cell stage, where normally prominent Xist ‘clouds’ covering the X chromosome are detected. We restricted the analysis to female embryos, which were identified based on the presence of two RNA-FISH signals for nascent Huwe1 RNA, an X-linked gene that is still expressed from both alleles at the eight-cell stage. Due to a developmental delay induced by the deletion, less Gata1/4/6^TKO^ embryos could be analysed than controls. We nevertheless observed a striking phenotype in the Gata1/4/6^TKO^ embryos, which showed generally very weak Xist signals and even absence of Xist upregulation in a subset of cells (Fig. 5f, Extended Data Fig. 7a). Quantification of Xist signals through automated image analysis revealed that Xist signal intensity was strongly reduced compared to control embryos (Fig. 5g, Extended Data Fig. 7b). These observations suggest that GATA factors, produced by the embryo, might be required for initial upregulation of Xist after fertilization. Given the strong reduction of Xist expression upon loss of GATA TFs, the absence of GATA factors in the pluripotent epiblast (Fig. 5b) might contribute to Xist downregulation at that stage.

### GATA-bound enhancers mediate Xist upregulation in vivo

Since the zygotic deletion of three GATA TFs did not only lead to reduced Xist expression, but also impaired the progression of embryonic development, we could not fully exclude the possibility that impaired Xist upregulation was an indirect consequence of the developmental delay. We therefore aimed at investigating more directly the role of GATA-bound elements in early Xist upregulation. We first tested whether the RE79 element, which drove GATA-dependent Xist upregulation in mESCs (see above), is part of the *tg80* and *tg53* single-copy transgenes, which can drive Xist expression in preimplantation embryos, but not in somatic cells^18,19^. RE79 is located around the telomeric end of the transgenes, but the precise extent has never been mapped (Extended Data Fig. 7c). We therefore performed quantitative PCR on genomic DNA from mESCs derived from the *tg80* and *tg53* mouse lines. We found that RE79 is indeed part of *tg80* and *tg53* (Extended Data Fig. 7d), which might thus allow GATA factors to drive Xist expression from the transgene.

**Fig. 6:**
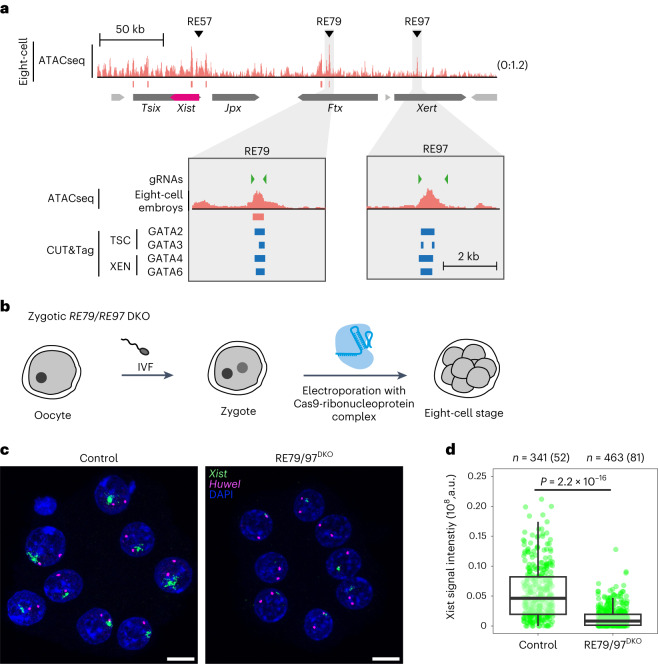
GATA-binding elements RE79 and RE97 are required for initial Xist upregulation in vivo. **a**, DNA accessibility measured by ATAC-seq in eight-cell stage mouse embryos^58^, showing open chromatin at GATA-bound Xist-regulatory elements RE79 and RE97. Green triangles show location of gRNA sequences used in **b**–**d**. **b**–**d**, Zygotic DKO of RE79 and RE97. **b**, Schematic depiction of the experimental workflow, where zygotes, generated by IVF were electroporated with Alt-R CRISPR/Cas9 ribonucleoprotein complex pre-assembled with four crRNAs targeting RE79 and RE97, as shown in **a** (green triangles). Embryos were allowed to develop to the eight-cell stage. **c**, RNA-FISH for Xist and the X-linked Huwe1 gene (nascent transcript) at the eight-cell stage. Only female embryos (two Huwe1 signals) were included in the analysis. In **d** the summed fluorescence intensity within the automatically detected Xist cloud is shown for individual cells. Embryos from two biological replicates were pooled (individual replicates are shown in Extended Data Fig. 7f). Statistical comparison was performed with a two-sided Wilcoxon rank-sum test. The central mark indicates the median, and the bottom and top edges of the box indicate the first and third quartiles, respectively. The top and bottom whiskers extend the boxes to a maximum of 1.5 times the interquartile range; cell (embryo) numbers are indicated on top. The scale bars in **c** represent 10 μm. Source numerical data are available as source data. Source Data

To further examine the role of RE79 in early Xist regulation, we re-analysed a published data set, where accessible regions had been mapped through ATAC-seq in preimplantation embryos^58^. At the eight-cell stage an ATAC peak is detected at RE79, suggesting that GATA factors might bind this region also in vivo (Fig. 6a). Interestingly, also RE97, which is bound by GATA TFs in XEN and TS cells (Fig. 4a), is accessible at the eight-cell stage. To test the functional role of GATA-bound elements in vivo, we deleted both elements in mouse zygotes and analysed Xist expression again at the eight-cell stage (Fig. 6b). We generated RE79/97-double knock-out (DKO) embryos, by combining four guide RNAs flanking the two genomic regions (Fig. 6a, green triangles in zoom in) and compared the effect on Xist to embryos electroporated with GFP-targeting control guides (Fig. 6c). The Xist signal in female RE79/97^DKO^ embryos was strongly reduced compared to the controls, which was again confirmed by quantification of Xist signal intensity (Fig. 6d, Extended Data Fig. 7e,f). Therefore, RE79 and RE97 appear to act as important long-range enhancers of Xist expression during early development. Given that they are bound by GATA TFs in extra-embryonic lineages, we conclude that GATA TFs indeed drive initial Xist upregulation through direct binding to these regulatory elements. With the GATA family we have therefore identified essential tissue-specific Xist activators and propose a key role for them in governing the initiation of XCI in vivo.

## Discussion

In this work, we identify GATA TFs as potent Xist activators and reveal a central role of GATA-mediated Xist regulation during early development. We show that all six family members are able to induce ectopic Xist upregulation in mESCs. We identify distal enhancer elements that mediate GATA6-dependent Xist induction and are bound by different GATA factors in extra-embryonic lineages. Finally, we demonstrate that Xist upregulation is strongly impaired upon simultaneous deletion of three GATA TFs in mouse zygotes or upon deletion of two GATA-responsive long-range enhancer elements. Given that different subsets of GATA TFs are present in all Xist*-*expressing cells in preimplantation embryos, but absent from pluripotent cells, where Xist is downregulated, we propose a role for this TF family in controlling XCI patterns during early development.

From our results a more complete picture emerges of how XCI is regulated during early development. It has previously been suggested that the XCI pattern is mostly controlled through Xist repression by pluripotency factors, either through direct binding of a regulatory element within *Xist’s* first intron, or indirectly through activation of Xist’s repressive antisense transcript Tsix^20,21,33,59^. However, Tsix is not required for Xist repression in the epiblast^23,60^ and deletion of the intron 1 binding site alone or in combination with a *Tsix* mutation does not lead to de-repression of Xist in mESCs^61,62^. In light of our findings, these results can be explained by the absence of activating factors in mESCs. We demonstrate that GATA factors are needed for the first upregulation of Xist upon fertilization from the paternal X chromosome. Due to the fact that GATA TFs are expressed in a variety of combinations during preimplantation development and in extra-embryonic lineages, they almost certainly contribute to the maintenance of Xist expression in those cellular contexts. The only cell type in the preimplantation embryo that does not express any GATA TF are pluripotent epiblast cells^63–65^. At E4.5, the downregulation of GATA factors (GATA4, GATA6) coincides with the loss of Xist expression and reactivation of the X chromosome^8,9^. Meanwhile, iXCI is sustained in the extra-embryonic lineages, which maintain the expression of GATA factors. Our finding that all GATA TFs are strong Xist activators, when overexpressed in pluripotent stem cells, suggests that the loss of GATA expression is likely required for Xist downregulation. Because GATA factors are expressed in a wide variety of cell types, including the blood and the heart^44^, this mode of regulation might also be involved in maintaining Xist expression in somatic cells.

In mESCs a single enhancer element, namely RE79, located ~100 kb upstream of the *Xist* promoter mediates GATA-induced Xist upregulation. We have recently shown that this element does not control Xist at the onset of rXCI^17^. In extra-embryonic cell lines, by contrast, additional sites are bound by GATA TFs, most prominently RE97, which we have recently shown to also be involved in the onset of rXCI^17^. We show that joint deletion of RE79 and RE97 largely prevents Xist upregulation in early embryos. Distinct, partially overlapping sets of long-range elements thus govern Xist upregulation in the context of iXCI and rXCI. Tissue-specific expression of Xist therefore appears to be orchestrated by a series of distal enhancer elements, which respond to lineage-specific TFs, such as GATA4 and GATA6 in the primitive endoderm, GATA2 and GATA3 in the trophectoderm, and OTX2 and SMAD2/3 in the epiblast. These long-range elements can, however, only induce Xist expression, if the promoter-proximal region is not repressed either by the rodent-specific imprint or through the RNF12–REX1-axis, which helps prevent Xist upregulation in male cells.

Imprinted XCI in extra-embryonic tissues has evolved specifically in rodents. However, also in human embryos Xist is upregulated shortly after fertilization^66^. In contrast to mice, Xist is expressed from all X chromosomes in male and female preimplantation embryos, but does not yet initiate XCI^67,68^. Given that multiple GATA TFs are expressed during preimplantation development in human embryos^68–70^, it is tempting to speculate that biallelic XIST upregulation is a result of GATA-dependent activation that can act on both X chromosomes, as the maternal *XIST* locus is not imprinted in humans.

A commonly assumed regulatory principle is that ubiquitous expression is governed by broadly expressed TFs^71^. Our results unveil a conceptually different regulatory strategy for ubiquitous expression: members of a TF family are expressed in specific cell types, yet together covering many different tissues. In this way, a group of TFs with tissue-specific expression patterns, but overlapping DNA binding preferences, would jointly drive near-ubiquitous expression of a target gene. Ongoing efforts to precisely map the transcriptome across tissues, such as the human cell atlas, will allow us to understand how common this regulatory strategy is used to shape gene expression in complex organisms.

## Methods

### Cell lines

The female TX1072 (clone A3), TX-SP106 (Clone D5) and TX-SP107 (Clone B6) mESC lines as well as the male E14-STN_ΔTsixP_ mESC cell line were described previously^17^. Briefly, the female TX1072 cell line (clone A3) is an F1 hybrid ESC line derived from a cross between the 57BL/6 (B6) and CAST/EiJ (Cast) mouse strains that carries a doxycycline-responsive promoter in front of the *Xist* gene on the B6 chromosome. TX1072 XO (clone H7/A3) is an XO line that was subcloned from TX1072 and has the B6 X chromosome. The TX-SP106 (Clone D5) mESC line stably expresses PYL1-VPR-IRES-Blast and ABI-tagBFP-SpdCas9, constituting a two-component CRISPRa system, where dCas9 and the VPR activating domain are fused to ABI and PYL1 proteins, respectively, which dimerize upon treatment with abscisic acid (ABA). The TX-SP107 (Clone B6) mESC line stably expresses PYL1-KRAB-IRES-Blast and ABI-tagBFP-SpdCas9, constituting a two-component CRISPRi system, where dCas9 and the KRAB repressor domain are fused to ABI and PYL1 proteins, respectively, which dimerize upon ABA treatment. Because repression in TX-SP107 cells transduced with sgRNAs was often observed already without ABA treatment, we could not make use of the inducibility of the system. Instead, TX-SP107 cells were always treated with ABA (100 µM) 72 h before the analysis and effects were compared to NTC sgRNAs. The male E14-STN_ΔTsixP_ mESC cell line expresses the CRISPRa SunTag system^28,29^ under a doxycycline-inducible promoter and carries a 4.2 kb deletion around the major *Tsix* promoter (ChrX: 103445995–103450163, mm10).

Female XEN XX #12 cell line was derived from a crossing of C57BL/6 (B6) female mice with CAST/Eij (Cast) males and were a kind gift from the Gribnau lab^72^. NGS karyotyping detected trisomies of chromosomes 1, 14 and 16. The female TSC line was derived from the CD1 mouse strain and was a kind gift from the Zernicka–Goetz lab. Low-passage HEK293T cells were a kind gift from the Yaspo lab. Details on all cell lines are given in Supplementary Table 4. All cell lines were routinely checked for XX status via RNA-FISH using a BAC probe for *Huwe1* as described below.

### mESC culture and differentiation

All mESC lines were grown on 0.1% gelatin-coated flasks in serum-containing medium (DMEM (Sigma), 15% ESC-grade FBS (Gibco), 0.1 mM β-mercaptoethanol), either supplemented with 1,000 U ml^–1^ LIF (Millipore) only (E14-STN_Δ__TsixP_, TX-SP106) or with LIF and 2i (3 μM Gsk3 inhibitor CT-99021, 1 μM MEK inhibitor PD0325901, Axon) (TX-SP107, TX-SP107-ERT2-Gata6-HA). Differentiation was induced by LIF or LIF/2i withdrawal in DMEM supplemented with 10% FBS and 0.1 mM β-mercaptoethanol at a density of 4–4.2 × 10^4^ cells/cm^2^ in fibronectin-coated (10 μg ml^–1^) tissue culture plates.

In CRISPRa-SunTag (E14-STN_Δ__TsixP_) experiments, the cells were treated with doxycycline (1 µg ml^–1^) for 3 days before harvesting. In CRISPRi and CRISPRa-VPR (TX-SP106) experiments, the cells were treated with Abscisic acid (ABA, Sigma 100 µM) for 3 days before harvesting. For nuclear translocation of ERT2-Gata6-HA, the cells were treated with 4OHT (Sigma, 2.5 μM).

### XEN and TS cells culture

Female XEN cell line was grown on 0.2% gelatin-coated flasks following the Rossant lab XEN stem cell protocol (https://lab.research.sickkids.ca/rossant/wp-content/uploads/sites/12/2015/08/XEN-Stem-Cell-protocol1.pdf) in serum-containing XEN medium (RPMI 1640 (Sigma, M3817)), 15% ESC-grade FBS (Gibco), 0.1 mM β-mercaptoethanol (Sigma), 1 mM sodium pyruvate (Gibco) and 2 mM l-glutamine (Life Technologies).

Female TSCs were grown on MEFs in serum-containing TSC medium (RPMI, 20 % fetal bovine serum, 1 mM sodium pyruvate, 100 mM 2-mercaptoethanol, 50 µg ml^–1^ penicillin/streptomycin and 2 mM l-glutamine; FGF4 (25 ng ml^–1^, R&D System) and Heparin (1 µg ml^–1^, Sigma) were added to the medium fresh prior to each use)^11^. Before sample collection, TSCs were passaged at least twice without MEFs to dilute out feeder cells. During this time cells were cultured in MEF-conditioned medium (70% MEF-conditioned medium, 30% TSC medium, FGF4 (37.5 ng ml^–1^, R&D System), Heparin (1.5 µg ml^–1^, Sigma)).

### Generation of transgenic cell lines

Transgenic cell lines were generated via lentiviral transduction. To package lentiviral vectors into lentiviral particles, 1 × 10^6^ HEK293T cells were seeded into one well of a six-well plate and transfected the next day with the lentiviral packaging vectors: 1.2 µg pLP1, 0.6 µg pLP2 and 0.4 µg pVSVG (Thermo Fisher Scientific), together with 2 µg of the desired construct using Lipofectamine 2000 (Thermo Fisher Scientific). HEK293T supernatant containing the viral particles was harvested after 48 h. 0.1–0.2 × 10^6^ mESCs were seeded per well in a 24–12-well plate in conventional ESC medium and transduced the next day with 0.25–0.5 ml of 10:1 concentrated (lenti-X, Clontech) supernatant with 8 ng µl^–1^ polybrene (Sigma Aldrich). Transgenic cells were selected with puromycin (sgRNA plasmids) (1 ng µl^–1^, Sigma) or hygromycin (FIREWACh plasmids, 200 ng µl^–1^, VWR) starting 2 days after transduction. Selection was kept for the entire experiment.

Cell lines overexpressing Gata1-6, Xist, Esx1, Cdx4 and Nup62cl via the CRISPRa SunTag system were generated by lentiviral transduction of E14-STN_ΔTsixP_ cells with sgRNAs, as indicated in the respective figure legend, targeted to the respective promoters or NTCs (Supplementary Table 4).

TX-SP107 CRISPRi cell lines for Gata1, Xist and REs (RE57/RE79/RE97) were generated by lentiviral transduction of TX-SP107/ TX-SP107-ERT2-Gata6-HA cells, carrying an ABA-inducible dCas9-KRAB system with plasmids carrying 1 (Xist) or 3 or 4 (Gata1/REs) sgRNAs targeted to the respective genomic loci or NTCs (SP125_LR249, SP199_mgLR9, SP199_mgLR22/23, SP199_mgVS012, SP199_mgLR15/16/17).

Cell lines expressing the FIREWACh reporter plasmid^56^ with the Gata RE regions and over-expressing Gata6 via the CRISPRa-ABA-inducible VPR system were generated by two rounds of lentiviral transduction. First, TX-SP106 (Clone D5) cells were transduced with plasmids carrying multi-sgRNAs targeting the *Gata6* promoter or NTCs (SP199_mgLR7, SP199_mgLR15/16). Then, either the empty (SP307) or the RE-containing FIREWACh plasmids (SP379, SP376, SP418) were lentivirally integrated into the cells, which were treated with abscisic acid (ABA, Sigma 100 µM) for 3 days before harvesting.

### Generation of KO mouse embryos

All animal procedures were conducted as approved by the local authorities (LAGeSo Berlin) under licence number G0243/18-SGr1. Oocytes were obtained from donor B6D2F1 female mice of 7–9 weeks of age (Envigo) by superovulation; hormone priming with 5 IU of PMSG followed by 5 IU of HCG 46 h later. 12 h after hormone priming, MII stage oocytes were isolated and cultured in standard KSOM media. Zygotes for knock-out experiments were obtained by performing in vitro fertilization (IVF) with donor oocytes and sperm under standard conditions. Sperm used for IVF is prepared from fertile F1 males (B6/CAST) as previously described^73^. Electroporation was performed as previously described^73^ with pre-assembled Alt-R CRISPR/Cas9 ribonucleoprotein complex (IDT). For the Gata1/Gata4/Gata6 TKO, three guides targeting exons were used for every target gene, for RE79/97 DKO guides were designed for sites flanking RE79 and RE97. Guide RNA sequences used can be found in Supplementary Table 4. Zygotes electroporated with a mock guide (targeting GFP) were used as control. Electroporated embryos were washed and cultured in KSOM medium in vitro under standard conditions (5% CO_2_, 37 °C). Gata1/4/6^TKO^ embryos developed slower than the controls.

### Flow cytometry

For Flow-FISH, the PrimeFlow RNA assay (Thermo Fisher) was used according to the manufacturer’s recommendations. Specifically, the assay was performed in conical 96-well plates with 5 × 10^6^ cells per well with Xist-specific probes, labelled with Alexa-Fluor647 (VB1-14258) (Thermo Fisher Scientific). Samples were resuspended in PrimeFlow RNA Storage Buffer before flow cytometry.

Flow cytometry data was collected using a BD FACSAria II, BD FACSAria Fusion or BD FACS Celesta flow cytometer. The sideward and forward scatter areas were used to discriminate cells from cell debris, whereas the height and width of the sideward and forward scatter were used for doublet discrimination. At least 30,000 cells were measured per sample. FCS files were analysed using RStudio with the flowCore (v1.52.1) and openCyto packages (v1.24.0)^74,75^.

For Flow-FISH, all cells that showed a fluorescence intensity above the 99th percentile of the undifferentiated cell population control, which does not express Xist, were marked as Xist-positive. These cells were then used to calculate the geometric mean in the Xist-positive fraction after background correction by subtracting the geometric mean of the undifferentiated control. In the enhancer–reporter assay, the geometric mean of the GFP fluorescence intensity was calculated and background-corrected by subtracting the geometric mean of the TX-SP106 non-transduced control (GFP negative).

### Molecular cloning

#### sgRNA cloning

To facilitate diagnostic digestion after cloning, an AscI restriction site was added to the original pU6-sgRNA-EF1*a*-puro-T2A-BFP plasmid (Addgene #60955^76^) between the BlpI and BstXI sites, resulting in plasmid SP125, by annealing the oligos LR148/LR149 that contain the restriction site. Single sgRNAs for CRISPRa were cloned into a BlpI and BstXI digested pU6-sgRNA-EF1*a*-puro-T2A-BFP plasmid by annealing oligos containing the guide sequence and recognition sites for BlpI and BstXI (Oligo F: 5′-TTGGNNN…NNNGTTTAAGAGC-3′and Oligo R: 5′-TTAGCTCTTAAACNNN…NNNCCAACAAG-3′) and ligating them together with the linearized vector using the T4 DNA ligase enzyme (NEB). Cloning of sgRNAs in a multiguide expression system (SP199) was performed as described previously^40^. Briefly, three or four different sgRNAs targeting the same gene/RE (Supplementary Table 4) were cloned into a single sgRNA expression plasmid with Golden Gate cloning, such that each sgRNA was controlled by a different Pol III promoter (mU6, hU6 hH1, h7SK) and fused to the optimized sgRNA constant region^77^. The vector (SP199) was digested with BsmBI (New England Biolabs) 1.5 h at 55 °C and gel-purified. Three fragments containing the optimized sgRNA constant region coupled to the mU6, hH1 or h7SK promoter sequences were synthesized as gene blocks (IDT). These fragments were then amplified with primers that contained part of the sgRNA sequence and a BsmBI restriction site (primer sequences can be found in Supplementary Table 4) and PCR-purified using the gel and PCR purification kit (Macherey & Nagel). The vector (100 ng) and two (for cloning three sgRNAs) or three (for cloning four sgRNAs) fragments were ligated in an equimolar ratio in a Golden Gate reaction with T4 ligase (New England Biolabs) and the BsmBI isoschizomer Esp3I (New England Biolabs) for 20 cycles (5 min 37 °C, 5 min 20 °C) with a final denaturation step at 65 °C for 20 min. Vectors were transformed into NEB Stable competent *E.* *coli*. Successful assembly was verified by ApaI digest and Sanger sequencing.

#### ERT2-Gata6-HA-T2A-Hygro overexpression construct

The plasmid was generated by standard molecular cloning techniques and its sequence is provided in the supplemental material (Supplementary Table 5). In brief, to generate ERT2-Gata6-HA-T2A-Hygro (SP299), the backbone of pLenti-ERT2-FLAG-Gal4-NLS-VP16-P2A-Puro (SP265) was used. SP265 was digested with NdeI/MluI (New England Biolabs) to remove FLAG-Gal4-NLS-VP16-P2A-Puro. The backbone was ligated with a HA-T2A-HygroR fragment, that was amplified from lenti-MS2-p65-HSF1_Hygro plasmid (Addgene #61426) using a primer that contained the HA-tag sequence via InFusion cloning. *Gata6* cDNA was PCR amplified from pSAM2_mCherry_Gata6 (Addgene #72694) and ligated using InFusion cloning.

#### Cloning of the Gata REs into the FIREWACh enhancer plasmid

To generate RE-containing enhancer–reporter plasmids, each RE (RE57, RE79 and RE97) was PCR-amplified from BAC (RP23-11P22, RP23-423B1) or genomic DNA with overhangs for InFusion cloning (Takara). The fragments were ligated into a BamHI digested FIREWACh plasmid FpG5 (Addgene #69443)^56^, to yield plasmids SP379, SP376, SP418.

FIREWACh RE In-Fusion cloning (Takara) was carried out in a 2:1 insert/vector ratio.

### RNA extraction, reverse transcription, qPCR

For gene expression profiling, cells were washed and lysed directly in the plate by adding 500 µl of Trizol (Invitrogen). RNA was isolated using the Direct-Zol RNA Miniprep Kit (Zymo Research) following the manufacturer’s instructions with on-column DNAse digestion. For quantitative RT-PCR (qRT-PCR), up to 1 µg RNA was reverse transcribed using Superscript III Reverse Transcriptase (Invitrogen) with random hexamer primers (Thermo Fisher Scientific) and expression levels were quantified in the QuantStudio 7 Flex Real-Time PCR machine (Thermo Fisher Scientific) using Power SYBR Green PCR Master Mix (Thermo Fisher Scientific) normalizing to Rrm2 and Arp0. Primers used are listed in Supplementary Table 4.

### RNA FISH on embryos

To prepare preimplantation embryos (eight-cell stage) for RNA-FISH, embryos were washed through a series of KSOM drops (Sigma), followed by a series of Tyrode’s solution. Zona pellucida was removed by incubating the embryos in Tyrode’s solution (Sigma) for 10–30 sec until the zona was dissolved. The embryos were washed through a series of PBS + 0.4% BSA prior to mounting onto poly-l-lysine (Sigma) coated (0.01% in H_2_O, 10 min incubation at room temperature) coverslip #1.5 (1 mm). Embryos were allowed to attach for about 2 min after which excess volume was removed and allowed to dry for 30 min. Embryos were fixed in 3% paraformaldehyde in PBS for 10 min at room temperature and permeabilized for 4 min on ice in PBS containing 0.5% Triton X-100 and 2 mM vanadyl-ribonucleoside complex (New England Biolabs). Coverslips were stored in 70% EtOH in –20 °C no longer than 1 day before further processing.

RNA-FISH was performed using the plasmid probe *p510* spanning the genomic sequence of *Xist* and the BAC probe (RP24-157H12) for *Huwe1* as described previously with minor modifications^78^. Both probes were labelled by nicktranslation (Abbot) with dUTP-Green (Enzo) or dUTP-Atto550 (Jena Bioscience), respectively. Per coverslip, 120–200 ng of each probe were ethanol precipitated (Cot1 repeats were included for *Huwe1* in order to suppress repetitive sequences in the BAC DNA that could hamper the visualization of specific signals), resuspended in 3-6 µl formamide and denatured (10 min 75 °C). For *Huwe1*, a competition step of 1 h at 37 °C was added. Before incubation with the probe, the samples were dehydrated through an ethanol series, 90% and 100%, twice of each (5 min each wash), and subsequently air-dried. Probes were hybridized in a 12 µl hybridization buffer overnight (50% Formamide, 20% Dextran Sulfate, 2x SSC, 1 µg/µl BSA, 10 mM Vanadyl-ribonucleoside). To reduce background, three 5 min washes were carried out in 50% Formamide/2× SSC (pH 7.2) and one 5 min wash in 2× SSC at 42 °C. Two additional washes in 2× SSC were carried out at room temperature and 0.2 mg ml^–1^ DAPI was added to the first wash. The samples were mounted using Vectashield mounting medium (Vector Laboratories).

Embryo image acquisition was performed using an inverted laser scanning confocal microscope with Airyscan (LSM880, Zeiss) with a 63×/1.4 NA oil objective, lateral resolution of 0.07 μm and 0.28 μm *Z*-sections in Fast Airyscan mode. Acquisition was performed under Zeiss ZEN 2.6 black software.

#### Automated analysis of RNA-FISH in embryos

Confocal *Z*-stacks were 3D airyprocessed using ZEN 2.6 Black and all subsequent analyses were performed in ZEN 3.2 or Zen 3.4 blue (both Zeiss) equipped with the Image Analysis module. The sex of each embryo was determined visually based on the RNA-FISH signal for the nascent transcript for *Huwe1*, an X-linked gene that is not yet silenced by XCI at the stages analysed (two signals per nucleus in females, one in males). Only female embryos were included in the analysis. Images were maximum intensity projected and a spot detector was used to identify primary objects (nuclei) by Gaussian smooth, Otsu-thresholding, dilation and water shedding. The resulting objects were filtered by area of 100–450 µm^2^ and circularity (sqrt((4 × area)/(π × FeretMax^2^))) of 0.7–1. Xist clouds were identified as a subclass within primary objects. Here, images were smoothed, background-subtracted (rolling ball), followed by a fixed intensity threshold to identify spots. Only nuclei with a Huwe1 signal were included in the downstream analysis. The summed signal intensity within the identified Xist spots were compared between cells in wildtype and TKO embryos using a Wilcoxon rank-sum test. Since the TKO embryos exhibited a developmental delay, less eight-cell embryos could be analysed compared to the control.

### Immunofluorescence combined with RNA FISH

IF-RNA-FISH was performed according to the Stellaris protocol for adherent cells, https://www.protocols.io/view/Stellaris-RNA-FISH-Sequential-IF-FISH-in-Adherent-ekzbcx6 with minor modifications. TX-SP107-ERT2-Gata6-HA cells as well as the parental TX-SP107 cell line were grown under 2i/LIF conditions. Two days before fixation, the cells were plated on fibronectin-coated coverslips (18 mm, Marienfeld) at a density of 2 × 10^4^ cells cm^–2^ in medium without 2i, which helps cells to spread sufficiently for imaging. Cells were fixed in 3% paraformaldehyde in PBS for 10 min at room temperature and permeabilized for 5 min at room temperature in PBS containing 0.1% Triton X-100, after 6 h and 24 h of 2.5 μM 4OHT treatment or after 48 h of LIF withdrawal as applicable. The coverslips were incubated with an HA-specific antibody (Abcam, ab9110 1:1,000) in PBS for 1 h at room temperature, then washed three times for 10 min with PBS, followed by a 1 h incubation with an Alexa-555 labelled Goat anti-rabbit antibody (Invitrogen A-21428, 0.8 μg ml^–1^). After three washes, the cells were fixed again with 3% paraformaldehyde in PBS for 10 min at room temperature, followed by three short washes with PBS and two washes with 2× SSC. Xist was detected using Stellaris FISH probes (Biosearch Technologies). Coverslips were incubated for 5 min in wash buffer containing 2× SSC and 10% formamide, followed by overnight hybridization at 37 °C with 250 nM of FISH probe in 50 μl Stellaris RNA FISH Hybridization Buffer (Biosearch Technologies) containing 10% formamide. Coverslips were washed twice for 30 min at 37 °C with 2× SSC/10% formamide with 0.2 mg ml^–1^ DAPI being added to the second wash. Prior to mounting with Vectashield mounting medium coverslips were washed with 2× SSC at room temperature for 5 min. Details on the antibodies and probes used are found in Supplementary Table 4.

Cell images were acquired using a widefield Axio Observer Z1/7 microscope (Zeiss) using a 100× oil immersion objective (NA = 1.4). Image analysis was carried out using Zen 3.1 blue (Zeiss). For each sample and replicate five tile regions were defined, the optimal focus was adjusted manually. The focused image was used as a centre for a *Z*-stack of 62 slices with an optimal distance of 0.23 µm between individual slices. Thereby, a total stack height of 14.03 µm was achieved covering slightly more than the cell height to ensure capturing of all events.

#### Automated analysis of IF-RNA-FISH

Image analysis was performed with ZEN 3.2 and 3.4 (Carl Zeiss, Germany). Images underwent a maximum intensity projection (MIP) of the full *Z*-stack of 62 slices. Segmentation of DAPI-stained nuclei was achieved with a priori trained Intellesis model. The identified objects were only kept in the subsequent steps, if they exhibited a circularity (Sqrt(4 × area/π × FeretMax^2^)) of 0.5–1 and an area of 50–300 µm^2^. Around each nucleus a ring (width 30 pix = 2.64 µm) was drawn and used as a surrogate for the cytoplasmic region. From the nuclear and cytoplasmic compartments the mean fluorescence intensity was extracted for the Gata6-HA staining and the nuclear-to-cytoplasmic ratio was calculated as a proxy for nuclear translocation. For identification of nuclear Xist signals, images were Gaussian smoothed, followed by a rolling ball background subtraction (radius 20 pixel) and a fixed intensity threshold. The identified areas were filtered to fit a circularity between 0.5 and 1. To quantify the Xist signal intensity the RNA-FISH signal was summed up within the segmented Xist signal. All cells with more than two Xist objects were excluded from the analysis.

### Immunofluorescence staining

Embryos were washed through a series of KSOM drops (Sigma), followed by a series of PBS + 0.4% BSA. Fixation was performed by incubation with 4% PFA for 15 mins. PFA was washed off by a series of washes in PBS + 0.5% TritonX-100 (PBS-T). Embryos were permeabilized in PBS-T for 20 min at room-temperature. After permeabilization, samples were washed in PBS-T and blocked in PBS-T + 2% BSA + 5% goat serum for 1 h at room temperature. Primary antibodies were diluted in blocking buffer (PBS-T + 2% BSA + 5% goat serum) overnight at 4 °C. Following incubation with the primary antibody (1:200), samples were washed three times for 10 min at room temperature in PBS-T + 2% BSA and subsequently incubated with secondary antibodies (1:1,000) in PBS-T + 2% BSA + 5% goat serum for 1 h at room temperature. Samples were washed three times 10 min at room temperature in PBS-T. After the last washing step, embryos were transferred to mounting medium (Vectashield, H1200) and further to a glass slide (Roth) and sealed with a cover glass (Brand, 470820). Detailed information on the antibodies used is given in Supplementary Table 4. Images were acquired with ZEISS LSM880 microscope at 40× magnification. Images were processed with ImageJ. Background fluorescence was subtracted by using rolling ball radius method (ImageJ) with 50 pixels as threshold.

### Tg80 mapping

QPCR was performed on genomic DNA from IKE15-9TG80 and IKE14-2TG53 (XY-tg), carrying a single copy of YAC PA-2^19^ and E14-STN_ΔTsixP_ (reference XY DNA) using primer pairs detecting different positions within the *Ftx* genomic locus. QPCR measurements were normalized to amplification from an X-linked locus outside of the YAC region (LR621/622). By calculating the ratio of the relative expression between the two cell lines, each genomic position could be classified as either internal (ratio ~2) or external (ratio ~1) to the YAC region.

### CRISPRa screen

#### CRISPRaX sgRNA library design

To target protein- and non-protein-coding X-linked genes via CRISPRa, sgRNA sequences were extracted from the mouse genome-wide CRISPRa-v2 library^79^ and complemented with newly designed sgRNAs using the CRISPR library designer (CLD) software^80^. Using Ensembl release (corresponding to genome assembly mouse mm10) and FANTOM 5 CAGE data^81^, a list of all TSSs for expressed genes (read count > 0, based on bulk-RNA seq data for female mESCs in 2i/LIF and 36 h -2i/LIF conditions) was compiled. All newly designed sgRNAs were in-silico tested for off-target effects in other promoter regions (550 bp window upstream of a TSS). In total the library targets 2,695 TSSs on the X chromosome, corresponding to 757 genes. Each TSS was targeted by 6 sgRNAs in a window between 550 and 25 bp upstream of the TSS. In cases where two TSSs were in close proximity, the same guides were used to target different TSSs. Additionally, two verified sgRNAs for *Xist* and guides targeting a series of known X-linked Xist regulators (*Rnf12*, *Ftx*, *Jpx*), autosomal Xist regulators (*Nanog*, *Zfp42*, *Sox2*, *Myc*, *Klf4*, *Esrrb*, *Pou5f1*, *Prdm14*, *Ctcf*, *Yy1*, *Eed*, *Chd8*, *Kat8*, *Msl1*, *Msl2*, *Kansl3*, *Kansl1*, *Mcrs1*, *Dnmt1*)^20–24,33,34,47,82–86^, and Xist-interacting proteins (*Spen*, *Lbr*, *Saf-A*, *Hnrnpk*)^87–89^ were included in the CRISPRaX library as well as 200 NTCs. The final library contained 8973 sgRNAs, which targeted 780 genes. The library composition is provided in Supplementary Table 1.

#### Cloning of CRISPRaX sgRNA library

The CRISPRaX sgRNA library was cloned into SP125, a modified pU6-sgRNA EF1Alpha-puro-T2A-BFP (pLG1) sgRNA expression plasmid (Addgene #60955^76^) where an AscI restriction site was added between the BstXI and the BlpI sites that enabled diagnostic digestion after ligation for verification of positive colonies. The library was cloned following the Weissman lab protocol https://weissmanlab.ucsf.edu/CRISPR/Pooled_CRISPR_Library_Cloning.pdf. sgRNA sequences, G + 19 nt, were synthesized by CustomArray flanked with OligoL (CTGTGTAATCTCCGACACCCACCTTGTTG) and OligoR (GTTTAAGAGCTAAGCTGGCCTTTGCATGTTGTGGA) sequences. For library amplification, three PCR reactions (primer sequences in Supplementary Table 4, LR169/LR170) with approx. 5 ng of the synthesized oligo pool were carried out using the Phusion High Fidelity DNA Polymerase (New England Biolabs), with a total of 15 cycles and an annealing temperature of 56 °C. The three PCR reactions were pooled and the 84 bp amplicons were PCR purified on a Qiagen Minelute column.

1 µg of the amplified sgRNAs was digested with BstXI (Thermo Fisher Scientific) and Bpu1102I (BlpI, Thermo Fisher Scientific) overnight at 37 °C. The digest was run on a 20% native acrylamide gel following staining with SYBR Safe DNA Gel Stain (Invitrogen) for 15 min. The 33 bp DNA fragment was extracted from the gel according to the Weissman lab protocol above. One 20 µl ligation reaction using T4 ligase (New England Biolabs) was carried out using 0.9 ng of the gel-purified insert and 500 ng of the vector. The reaction was EtOH-precipitated to remove excess salts which might impair bacterial transformation and resuspended in 20 µl H_2_O. 8 µl of the eluted DNA were transformed into 20 µl of electrocompetent cells (MegaX DH10B, Thermo Fisher Scientific) according to the manufacturer’s protocol using the ECM 399 electroporator (BTX). After a short incubation period (1 h, 37 °C, shaking) in 1 ml SOC medium, 9 ml of LB medium with Ampicillin (0.1 mg/ml, Sigma) were added to the mixture and dilutions were plated in Agar plates (1:100, 1:1,000 and 1:10,000) to determine the coverage of the sgRNA library (2,000×). 500 ml of LB media with Ampicillin were inoculated with the rest of the mixture and incubated overnight for subsequent plasmid purification using the NucleoBond Xtra Maxi Plus kit (Macherey-Nagel) following the manufacturer’s instructions. To confirm library composition and even sgRNA representation by deep-sequencing a PCR reaction was carried out to add illumina adaptors and a barcode by using the Phusion High Fidelity DNA Polymerase (New England Biolabs), with an annealing temperature of 56 °C and 15 cycles (LR177/LR175, see Supplementary Table 4). The PCR amplicon was gel-purified by using the QIAquick Gel Extraction Kit (Qiagen) following the manufacturer’s instructions. Library was sequenced paired-end 75 bp on the HiSeq 4000 Platform using the sequencing primer LR176 yielding approximately 6 million fragments. Read alignment statistics found in Supplementary Table 1).

#### Viral packaging of sgRNA library

To package the CRISPRaX library into lentiviral particles, HEK293T cells were seeded into eleven 10 cm plates. The next day at 90% confluence each plate was transfected with 6.3 µg of pLP1, 3.1 µg of pLP2 and 2.1 µg of VSVG packaging vectors (Thermo Fisher Scientific) together with 10.5 µg of the CRISPRaX library plasmid in 1 ml of Opti-MEM (Life technologies) using 60 µl lipofectamine 2000 reagent (Thermo Fisher Scientific) according to the manufacturer’s instructions. After 48 h the medium was collected and centrifuged at 1,800*g* for 15 min at 4 °C. Viral supernatant was further concentrated 10-fold using the lenti-X^T^ Concentrator (Takara Bio) following the manufacturer’s instructions and subsequently stored at –80 °C.

To assess the viral titre, four serial 10-fold dilutions of the viral stock were applied to each well of a six-well mESC plate (MOCK plus 10^–3^ to 10^–6^) for transduction with 8 ng µl^–1^ polybrene (Merck). Two replicates were generated for each well. Selection with puromycin (1 ng µl^–1^, Sigma) was started 2 days after transduction and colonies were counted after 7 days. The estimated titre was 5.43 × 10^6^ transducing units (TU) per ml.

#### Transduction

For the CRISPRa-SunTag screen, male E14-STN_ΔTsixP_ mESCs were passaged twice before 1.2 × 10^7^ cells were transduced with the CRISPRaX sgRNA library (MOI = 0.3). Puromycin selection (1 ng µl^–1^, Sigma) was started 48 h after transduction and kept until the end of the experiment. Four days after transduction, 7.2 × 10^7^ cells were differentiated by LIF withdrawal for 2 days. Expression of CRISPRa-SunTag system was induced using doxycycline (Clontech, 1 µg ml^–1^) one day before differentiation and kept throughout the rest of the protocol. Cells were harvested with trypsin to reach a single-cell suspension for Flow-FISH after 2 days of differentiation.

#### Flow-FISH and cell sorting

Phenotypic enrichment based on RNA levels was performed as previously described^90^. The PrimeFlow RNA assay (Thermo Fisher) was used as described above. 2.4 × 10^8^ cells were stained, while 2 × 10^7^ cells were snap-frozen after the second fixation step to be used as the unsorted fraction. The 15% of cells with the highest fluorescence were sorted using a BD FACSAria II flow cytometer, recovering 7–15 × 10^6^ cells per replicate. After sorting, the cell pellet was snap-frozen and stored at –80 °C for further analysis.

#### Preparation of sequencing libraries and sequencing

Sequencing libraries were prepared from both sorted and unsorted cell populations. Genomic DNA from frozen cell pellets was isolated by Phenol/Chloroform extraction. Briefly, cell pellets were thawed and resuspended in 250 µl of Lysis buffer (1% SDS (Thermo Fisher Scientific), 0.2 M NaCl and 5 mM DTT (Roth) in TE Buffer) and incubated overnight at 65 °C. 200 µg of RNAse A (Thermo Fisher Scientific) were added to the sample and incubated at 37 °C for 1 h. 100 µg of Proteinase K (Sigma) were subsequently added followed by a 1 h incubation at 50 °C. Phenol/chloroform/isoamyl alcohol (Roth) was added to each sample in a 1:1 ratio, the mixture was vortexed for 1 min and subsequently centrifuged at 16,000*g* for 10 min at room temperature. The aqueous phase was transferred to a new tube, 1 ml 100% EtOH, 90 µl 5 M NaCl and 2 µl Pellet Paint (Merck) was added to each sample, mixed, and incubated at –80 °C for 1 h. DNA was pelleted by centrifugation for 16,000*g* for 15 min at 4 °C, pellets were washed twice with 70% EtOH, air-dried and resuspended in 50 µl H_2_O.

The genomically integrated sgRNA cassette was PCR-amplified to attach sequencing adaptors and sample barcodes. To ensure proper library coverage (300×), a total of 20 µg of each sample were amplified using the ReadyMix Kapa polymerase (Roche) with a total of 25 cycles and an annealing temperature of 56 °C. A relatively low amount of 0.5 µg genomic DNA was amplified per 50 µl PCR reaction since in samples stained with Flow-FISH, PCR amplification was inhibited at higher DNA concentrations. PCR was performed with the primer LR175 in combination with a sample-specific primer which contains a distinct six-nucleotide barcode to allow sample identification after multiplexed deep sequencing (Primer sequences in Supplementary Table 4, LR178/LR180). Successful amplification was verified on a 1% agarose gel and the reactions were pooled. 1 ml of each pooled PCR was purified using the QIAquick PCR Purification Kit (Qiagen), loaded on a 1% agarose gel and purified using the QIAquick Gel Extraction Kit (Qiagen).

Libraries were sequenced as follows: replicate 1, paired-end 75 bp on the HiSeq 4000 platform; replicate 2, paired-end 50 bp on the HiSeq 2500 platform; replicate 3, single-read 75 bp on the HiSeq 2500 platform, using the custom primer LR176 yielding approximately 8 × 10^6^ fragments per sample (read alignment statistics are shown in Supplementary Table 1).

#### Screen analysis

Data processing and statistical analysis was performed using the MAGeCK CRISPR screen analysis tools (v0.5.9.3)^31,32^. Alignment and read counting was performed with options [count–norm-method control]. At least 6.95 × 10^6^ mapped reads were obtained per sample. Correlation between the three replicates was computed as a Pearson correlation coefficient on the normalized counts (Extended Data Fig. 1d). The NTC distribution width was similar across samples, suggesting that sufficient library coverage was maintained during all steps (Extended Data Fig. 1e). Statistical analysis was performed in two steps. Since the CRISPRaX library often targets multiple TSSs per gene, with a subset of sgRNAs targeting multiple TSSs, we first identified one TSS per gene with the strongest effect. To this end, a first analysis was performed on the transcript level, including all TSS, with options [mle –norm-method control]. For each gene the TSS with the lowest Wald.fdr was identified. Then a statistical analysis was performed on the gene level, based on only those sgRNAs that targeted the identified TSS with options [mle–norm-method control]. Genes were ranked for their effect on Xist expression based on their beta score, a measure of the effect size estimated by the MAGeCK mle tool. For all visualization purposes the name Rnf12 was used for Rlim and Oct4 was used for Pou5f1. Alignment statistics, raw counts and gene hit summary files are provided in Supplementary Table 1.

### Bulk RNA-sequencing

Differentiating TX1072 XO mESCs (clone H7/A3) were profiled in three biological replicates by bulk RNA-seq as described previously for TX1072 XX mESCs^30^. RNA-seq libraries were generated using the Tru-Seq Stranded Total RNA library preparation kit (Illumina) with 1 µg starting material for rRNA-depletion and amplified with 15 cycles of PCR. Libraries were sequenced 2 × 50 bp on a HiSeq 2500 with 1% PhiX spike-in, which generated ~50 million fragments per sample.

### CUT&Tag

CUT&Tag experiments were performed on XEN and TS female cells as described previously^17^. Cells were washed with PBS and dissociated with accutase. For each CUT&Tag reaction 1 × 10^5^ cells were collected and washed once with wash buffer (20 mM HEPES-KOH, pH 7.5, 150 mM NaCl, 0.5 mM spermidine, 10 mM sodium butyrate, 1 mM PMSF). 10 μl Concanavalin A beads (Bangs Laboratories) were equilibrated with 100 μl binding buffer (20 mM HEPES-KOH, pH 7.5, 10 mM KCl, 1 mM CaCl_2_, 1 mM MnCl_2_) and afterwards concentrated in 10 μl binding buffer. The cells were bound to the Concanavalin A beads by incubating for 10 min at room temperature with rotation. Following this, the beads were separated on a magnet and resuspended in 100 μl chilled antibody buffer (wash buffer with 0.05% digitonin and 2 mM EDTA). Subsequently 0.5 μl (GATA2/3/4/6 and IgG control) or 1 μl (H3K27ac, H3K27me3) of primary antibody was added and incubated on a rotator for 3 h at 4 °C. After magnetic separation the beads were resuspended in 100 μl chilled dig-wash buffer (wash buffer with 0.05% Digitonin) containing 1 μl of matching secondary antibody (1:100) and were incubated for 1 h at 4 °C with rotation. The beads were washed three times with ice-cold dig-wash buffer and resuspended in chilled dig-300 buffer (20 mM HEPES-KOH, pH 7.5, 300 mM NaCl, 0.5 mM spermidine, 0.01% digitonin, 10 mM sodium butyrate, 1 mM PMSF) with 1:250 diluted 3×FLAG-pA-Tn5 preloaded with mosaic-end adapters. After incubation for 1 h at 4 °C with rotation, the beads were washed four times with chilled dig-300 buffer and resuspended in 50 μl tagmentation buffer (dig-300 buffer 10 mM MgCl_2_). Tagmentation was performed for 1 h at 37 °C and subsequently stopped by adding 2.25 μl 0.5 M EDTA, 2.75 ml 10% SDS and 0.5 μl 20 mg ml^–1^ Proteinase K and vortexing for 5 sec. DNA fragments were solubilized for 14 h at 55 °C followed by 30 min at 70 °C to inactivate residual Proteinase K. To remove the beads, the samples were put on a magnetic rack and the supernatants were transferred to a new tube. DNA fragments were purified with the ChIP DNA Clean & Concentrator kit (Zymo Research) and eluted with 25 μl elution buffer according to the manufacturer’s guidelines. Antibodies used can be found in Supplementary Table 4.

#### Library preparation and sequencing

NGS libraries were generated by amplifying 12 μl of the eluted CUT&Tag DNA fragments with i5 and i7 barcoded HPLC-grade primers^91^ (Supplementary Table 4) with NEBNextHiFi 2× PCR Master Mix (New England BioLabs) on a thermocycler with the following program: 72 °C for 5 min, 98 °C for 30 s, 98 °C for 10 s, 63 °C for 10 s (14–15 cycles for step 3–4) and 72 °C for 1 min. Post PCR cleanup was performed with Ampure XP beads (Beckman Coulter). For this 1.1× volume of Ampure XP beads were mixed with the NGS libraries and incubated at room temperature for 10 min. After magnetic separation, the beads were washed three times on the magnet with 80% ethanol and the libraries were eluted with Tris-HCl, pH 8.0. The quality of the purified NGS libraries was assessed with the BioAnalyzer High Sensitivity DNA system (Agilent Technologies). Sequencing libraries were pooled in equimolar ratios, cleaned again with 1.2× volume of Ampure XP beads and eluted in 20 μl Tris-HCl, pH 8.0. The sequencing library pool quality was assessed with the BioAnalyzer High Sensitivity DNA system (Agilent Technologies) and subjected to Illumina PE75 next generation sequencing on the NextSeq500 platform totalling 1–12 million fragments per library (see Supplementary Table 3 for details).

### NGS data analysis

#### Published ChIP-seq & ATAC-seq data

FASTQ files for TF binding data of FLAG-tagged GATA6 in mESCs after 36 hours of dox-mediated GATA6 overexpression^49^ was retrieved from the Gene Expression Omnibus (GEO) Accession Viewer (GSE69322) using fasterq-dump (v2.9.4) (http://ncbi.github.io/sra-tools/). FASTQ files for ATAC-seq data from eight-cell stage mouse embryos, was similarly acquired from the GEO Accession Viewer (GSE66581).

#### Data processing

For ATAC-seq, CUT&Tag and ChIP-seq data, reads were trimmed for adapter sequences using Trim Galore (v0.6.4) with options [–paired–nextera] for CUT&Tag/ATAC-seq or [–paired–illumina] for CHIP-seq (http://www.bioinformatics.babraham.ac.uk/projects/trim_galore/) prior to alignment. Read alignment was performed to the mm10 reference genome using bowtie2 (v2.3.5.1) with options [--local–very-sensitive-local–no-mixed–no-discordant –phred33 -I 10 -X 2000]^92^ for CUT&Tag/ChIP-seq and [--local–very-sensitive -X 2000] for ATAC-seq or with STAR (v2.7.5a) with options [–outSAMattributes NH HI NM MD]^93^ for RNA-seq. For ATAC-seq, mitochondrial reads were removed using a custom python script. Sequencing data was then filtered for properly mapped reads and sorted using samtools^94^ (v1.10) with options [view -f 2 -q 20] (ATAC-seq/CUT&Tag/ChIP-seq) or [view -q 7 -f 3] (RNA-seq) and [sort]. For ATAC-seq/ChIP-seq/CUT&Tag, blacklisted regions for mm10 (ENCODE Project Consortium, 2012) were removed using bedtools^95^ (v2.29.2) with options [intersect -v]. For ATAC-seq & ChIP-seq, reads were also deduplicated using Picard (v2.18.25) with options [MarkDuplicates VALIDATION_STRINGENCY = LENIENT REMOVE_DUPLICATES = TRUE] (http://broadinstitute.github.io/picard). Mapping statistics and quality control metrics for RNA-seq/CUT&Tag can be found in Supplementary Tables 2 and 3.

#### Generation of coverage tracks & Peak calling

BIGWIG coverage tracks for ATAC-seq, CUT&Tag and ChIP-seq were created using deeptools2 (v3.4.1)^96^ on merged replicates with the options [bamCoverage -bs 10 -e–normalizeUsing CPM -ignore chrX chrY]. The tracks were visualized using the UCSC genome browser^97^. Peaks were called using MACS2^98^ (v2.1.2) with standard options [callpeak -f BAMPE/BAM -g mm -q 0.05] on individual replicates. For ChIP-seq, input samples were included for normalization using [-c]. Only peaks detected in all replicates were retained by merging replicates using bedtools^95^ (v2.29.2) with [intersect].

#### Correlation analysis

For CUT&Tag, BAM files, excluding mitochondrial reads, were counted in 1 kb bins using deepTools2^96^ (v3.4.1) with options [multiBamSummary bins -bs 1000 -bl chrM.bed]. The Pearson correlation coefficient between different samples was then computed with options [plotCorrelation -c pearson]. The resulting values were hierarchically clustered and plotted as a heatmap (Extended Data Fig. 5b).

#### Annotation of GATA factor motifs within CUT&Tag peaks within the Xic

FASTA files containing the sequences of all GATA TF CUT&Tag peaks that were identified in both replicates were generated using bedtools (v2.29.2)^95^ with options [getfasta]. The FASTA files were scanned for the occurrence of the respective GATA TF binding motif, which were retrieved from the JASPAR database^99^ (8th release) using FIMO (v5.1.1) with options [–thresh 0.001]^100^. The location and annotation of all peaks within the *Xic* is shown in Supplementary Table 3.

#### Verification of GATA CUT&Tag data

To assess specificity of the identified peaks, we compared the intensity of peaks with a GATA motif to those without. To this end, we used RSubread (Liao et al., 2019) (v2.0.1) with options [featureCounts(isPairedEnd = TRUE)] to calculate Reads per Million (RPM) in peaks with or without a motif individually. Subsequently, we plotted their density (Extended Data Fig. 5c). While peaks with a motif were clearly stronger for GATA6, and to a slightly lesser extent also for GATA3 and GATA4, no difference was observed for GATA2 (Extended Data Fig. 5c).

Furthermore, we identified enriched motifs within all peaks of each CUT&Tag data set. We performed motif enrichment using the non-redundant vertebrate JASPAR2020 CORE position frequency matrix (PFM) data set, as described previously^101^ with adaptations. To this end, all peaks that were identified in both replicates were centred and extended to a total of 500 bp. Afterwards, Rsubread^102^ (v2.0.1) with options [featureCounts(isPairedEnd = TRUE)] was used to quantify the number of reads mapping to each peak. The centred peaks were ranked depending on RPM and transformed into FASTA files using bedtools (v2.29.2)^95^ with options [getfasta]. These files were scanned for enriched PFMs using AME (v5.1.1)^103^ with options [–shuffle]. For GATA3, GATA4 and GATA6 all top-ranking motifs were members of the GATA family, while no GATA motifs were found for GATA2. These analyses suggest that GATA3, GATA4 and GATA6 can be profiled reliably by CUT&Tag, while the data for GATA2 should be interpreted with caution. The complete results of the motif enrichment analysis are shown in Supplementary Table 3.

#### Gene quantification of RNA-seq data

RNA-seq data during the differentiation of female TX1072 mESCs (XX) was acquired from GSE151009^30^. (Single-cell)-RNA-seq data during mouse embryonic development^42,43^ was similarly acquired from GEO (GSE45719, GSE76505). The single-cell data was merged as a pseudo-bulk prior to alignment. Gene expression was quantified using the GENCODE M25 annotation^104^. Rsubread^102^ (v2.0.1) was used with the options [featureCounts(isPairedEnd = TRUE, GTF.featureType = ‘exon’, strandSpecific = 2)]. Transcripts per million reads (TPM) values for the XX and XO time courses can be found in Supplementary Table 3.

### Single-cell RNA-seq analysis

For reanalysis of previously published scRNA-seq data from mouse embryos, the normalized data from study of preimplantation embryos up to E3.5^42^ was downloaded from GEO (GSE45719) and data from E4.5–E6.5 embryos^57^ was downloaded from https://github.com/rargelaguet/scnmt_gastrulation together with the cell type annotation and visualized in R.

### Statistics and reproducibility

No statistical method was used to predetermine sample size. No data were excluded from the analyses. The experiments were not randomized. The investigators were not blinded to allocation during experiments and outcome assessment. Statistical analyses were conducted in R (v4.2.2), if not stated otherwise.

### Reporting summary

Further information on research design is available in the Nature Portfolio Reporting Summary linked to this article.

## Online content

Any methods, additional references, Nature Portfolio reporting summaries, source data, extended data, supplementary information, acknowledgements, peer review information; details of author contributions and competing interests; and statements of data and code availability are available at 10.1038/s41556-023-01266-x.

### Supplementary information

Reporting Summary

Supplementary Table 1 CRISPRaX sgRNA library and screen analysis. Related to Fig. 1 and Extended Data Fig. 1.

Supplementary Table 2 Bulk RNA-seq on TX1072 XX and XO cells. Related to Extended Data Fig. 2.

Supplementary Table 3 CUT&Tag analysis and AME motif discovery results. Related to Fig. 4 and Extended Data Fig. 5.

Supplementary Table 4 Cell lines, oligos, probes, antibodies used in this study.

Supplementary Table 5 Plasmid sequences.

### Source data

Source Data Figs. 1–6 and Extended Data Figs. 1–7 Statistical Source Data.

## Data Availability

Sequencing (CRISPRa screen, CUT&Tag and TX1072 XO bulk RNA-seq) data sets that support the findings of this study have been deposited in the GEO under accession numbers GSE194018. Previously published scRNA-seq, bulk RNA-seq, ATAC-seq and ChIP-seq data that were re-analysed here are available on GEO under accession codes GSE121708, GSE45719, GSE151009, GSE66581, GSE69323 and on GitHub (https://github.com/rargelaguet/scnmt_gastrulation). The JASPAR database^99^ (eighth release) is available at https://jaspar2020.genereg.net/downloads/. Source data are provided with this paper. All other data supporting the findings of this study are available from the corresponding author on reasonable request.

## Code availability

Custom code associated with the study is available at https://github.com/EddaSchulz/GATA_paper.
