# Supplementary material for: GATA transcription factors drive initial Xist upregulation after fertilization through direct activation of long-range enhancers
Source: Nat Cell Biol. 2023 Nov 6;25(11):1704–15. doi: 10.1038/s41556-023-01266-x (PMC10635832; doi:10.1038/s41556-023-01266-x)
Supplement: Supplementary file 1 — Reporting Summary [file 41556_2023_1266_MOESM1_ESM.pdf]

nature portfolio

Corresponding author(s): Edda Schulz

Last updated by author(s): 21/8/23

Reporting Summary

Nature Portfolio wishes to improve the reproducibility of the work that we publish. This form provides structure for consistency and transparency in reporting. For further information on Nature Portfolio policies, see our Editorial Policies and the Editorial Policy Checklist.

Statistics

For all statistical analyses, confirm that the following items are present in the figure legend, table legend, main text, or Methods section.

n/a	Confirmed	
☐	☒	The exact sample size (*n*) for each experimental group/condition, given as a discrete number and unit of measurement
☐	☒	A statement on whether measurements were taken from distinct samples or whether the same sample was measured repeatedly
☐	☒	The statistical test(s) used AND whether they are one- or two-sided<br>*Only common tests should be described solely by name; describe more complex techniques in the Methods section.*
☒	☐	A description of all covariates tested
☒	☐	A description of any assumptions or corrections, such as tests of normality and adjustment for multiple comparisons
☐	☒	A full description of the statistical parameters including central tendency (e.g. means) or other basic estimates (e.g. regression coefficient) AND variation (e.g. standard deviation) or associated estimates of uncertainty (e.g. confidence intervals)
☐	☒	For null hypothesis testing, the test statistic (e.g. *F*, *t*, *r*) with confidence intervals, effect sizes, degrees of freedom and *P* value noted<br>*Give P values as exact values whenever suitable.*
☒	☐	For Bayesian analysis, information on the choice of priors and Markov chain Monte Carlo settings
☒	☐	For hierarchical and complex designs, identification of the appropriate level for tests and full reporting of outcomes
☐	☒	Estimates of effect sizes (e.g. Cohen's *d*, Pearson's *r*), indicating how they were calculated

Our web collection on statistics for biologists contains articles on many of the points above.

Software and code

Policy information about availability of computer code

Data collection	Microscopy data was collected with Zen 3.1 blue (Zeiss)
Data analysis	Image analysis was performed with ZEN 3.2 and 3.4 (Carl Zeiss, Germany).<br>For NGS analysis the following tools were used: Trim Galore (0.6.4), bowtie2 (v2.3.5.1) , STAR (v2.7.5a), samtools (v1.10) ,bedtools v(v2.29.2), Picard (v2.18.25), deeptools2 (v3.4.1) , MACS2 (v2.1.2), RSubread (v2.0.1), fasterq-dump (v2.9.4), FIMO (v5.1.1), AME (v5.1.1), R (v.4.2.2)<br>Flow cytometry: R packages flowCore (v1.52.1), openCyto (v1.24.0)<br>Custom Code is available at https://github.com/EddaSchulz/GATA_paper

For manuscripts utilizing custom algorithms or software that are central to the research but not yet described in published literature, software must be made available to editors and reviewers. We strongly encourage code deposition in a community repository (e.g. GitHub). See the Nature Portfolio guidelines for submitting code & software for further information.

Data

Policy information about availability of data

All manuscripts must include a data availability statement. This statement should provide the following information, where applicable:

- Accession codes, unique identifiers, or web links for publicly available datasets
- A description of any restrictions on data availability
- For clinical datasets or third party data, please ensure that the statement adheres to our policy

CRISPRa screen, CUT&Tag and TX1072 XO bulk RNA-seq data sets are available via GEO (GSE194018). The scRNA-seq data from mouse embryos analyzed in this study42,57, are available on Github (https://github.com/rargelaguet/scnmt_gastrulation) and on GEO (GSE121708, GSE45719). Bulk RNA-seq for TX1072 XX 30, ATAC-seq for 8-cell stage embryos and ChIP-seq for FLAG-tagged GATA649 are available via GEO (GSE151009, GSE66581, GSE69323).

Human research participants

Policy information about studies involving human research participants and Sex and Gender in Research.

Reporting on sex and gender	No human subjects participated in the study.
Population characteristics	No human subjects participated in the study.
Recruitment	No human subjects participated in the study.
Ethics oversight	No human subjects participated in the study.

Note that full information on the approval of the study protocol must also be provided in the manuscript.

Field-specific reporting

Please select the one below that is the best fit for your research. If you are not sure, read the appropriate sections before making your selection.

☒ Life sciences ☐ Behavioural & social sciences ☐ Ecological, evolutionary & environmental sciences

For a reference copy of the document with all sections, see nature.com/documents/nr-reporting-summary-flat.pdf

Life sciences study design

All studies must disclose on these points even when the disclosure is negative.

Sample size	No statistical method was used to predetermine sample size. Most experiments were performed in duplicate or triplicate to assess whether the observed effects were reproducible.
Data exclusions	no data was excluded.
Replication	All observations were replicated at least 2 or 3 times
Randomization	No randomization was included in the study design, because the processing order does not effect the outcome in the molecular biology experiements performed here.
Blinding	No blining was included in the study design, because all data analysis was performed computationally.

Reporting for specific materials, systems and methods

We require information from authors about some types of materials, experimental systems and methods used in many studies. Here, indicate whether each material, system or method listed is relevant to your study. If you are not sure if a list item applies to your research, read the appropriate section before selecting a response.

Materials & experimental systems

n/a	Involved in the study
☐	☒ Antibodies
☐	☒ Eukaryotic cell lines
☒	☐ Palaeontology and archaeology
☐	☒ Animals and other organisms
☒	☐ Clinical data
☒	☐ Dual use research of concern

Methods

n/a	Involved in the study
☐	☒ ChIP-seq
☐	☒ Flow cytometry
☒	☐ MRI-based neuroimaging

Antibodies

Antibodies used	Gata1 Cell signaling 3535, 1:200<br>Gata2 Abcam ab109241, 1:200<br>Gata3 BD Pharmingen 558686, 1:200<br>Gata4 Santa Cruz sc-25310, 1:200<br>Gata6 R&D Systems AF1700, 1:200<br>H3K27me3 Active Motif 61017, 1:100<br>H3K27ac Active Motif 39685, 1:100<br>HA Abcam ab9110, 1:200<br>Guinea Pig anti-rabbit IgG Antibodies Online ABIN101961, 1:100<br>Rabbit anti-goat IgG Thermo Fisher Scientific A27011, 1:100<br>rabbit anti-mouse IgG Invitrogen 31194, 1:100<br>Donkey anti-mouse Alexa 488 Invitrogen A21202, 1:1000<br>Donkey anti-rabbit Alexa 647 Jackson Immuno 711605152, 1:1000<br>Donkey anti-goat 594 Invitrogen A11058, 1:1000<br>Alexa-555 labeled Goat anti-rabbit antibody Invitrogen A-21428, 0.8 ug/ml
Validation	Gata1, Gata2, Gata3, Gata4 and Gata6 antibodies have been validated through gene knock-out which leads to absence of the signal. For the H3K27me3 and H3K27Ac antibodies dot blot analysis was used to confirm the specificity by the supplier. For the HA antibody specificity was confirmed by absence of signal, when no HA-tagged protein was expressed.

Eukaryotic cell lines

Policy information about cell lines and Sex and Gender in Research

Cell line source(s)	The female TX1072 cell line and its subclone TX1072 XO (clone A11) were previously derived in Edith Heard's lab by Edda Schulz (Schulz et al, Cell Stem Cell, 2014). The cell lines were provided by Edith Heard (Institut Curie). The E14 SunTag cell line (E14-STN) was provided by Pablo Navarro (Institut Pasteur) (Heurtier et al, Nat Comm, 2019) and the XEN cell line was provided by Joost Gribnau (Erasmus Medical Center Rotterdam). The CD1-derived female TSC line was provided by Magdalena Zernicka-Goetz (Caltech). Low-passage Hek293T cells were provided by Marie-Laure Yaspo (MPI for Mol Gen).
Authentication	The cell lines used were not authenticated. The number of X chromosomes was regularly checked by RNA FISH for X-linked genes.
Mycoplasma contamination	Cells were regularly tested for mycoplasma contamination, test results were always negative.
Commonly misidentified lines<br>(See ICLAC register)	No such cell lines were used in this study.

Animals and other research organisms

Policy information about studies involving animals; ARRIVE guidelines recommended for reporting animal research, and Sex and Gender in Research

Laboratory animals	Mouse embryos were generated by in vitro fertiliation, using oocytes obtained from donor B6D2F1 female mice of 7-9 weeks of age (Envigo) by superovulation, fertilized with sperm from F1 males (B6/CAST).
Wild animals	No wild animals were used in this study
Reporting on sex	Analysis was restricted to female embryos, since only those undergo X-chromosome inactivation.
Field-collected samples	No field-collected samples were used in this study
Ethics oversight	All animal procedures were conducted as approved by the local authorities (LAGeSo Berlin) under the license number G0243/18-SGr1.

Note that full information on the approval of the study protocol must also be provided in the manuscript.

ChIP-seq

Data deposition

☒ Confirm that both raw and final processed data have been deposited in a public database such as GEO.

☒ Confirm that you have deposited or provided access to graph files (e.g. BED files) for the called peaks.

Data access links<br>*May remain private before publication.*	https://www.ncbi.nlm.nih.gov/geo/query/acc.cgi?acc=GSE194018
Files in database submission	TS_XX_GATA2_r1.bw<br>TS_XX_GATA2_r2.bw<br>TS_XX_GATA3_r1.bw<br>TS_XX_GATA3_r2.bw<br>TS_XX_H3K27ac_r1.bw<br>TS_XX_H3K27ac_r2.bw<br>TS_XX_H3K27me3_r1.bw<br>TS_XX_H3K27me3_r2.bw<br>XEN_XX_GATA4_r1.bw<br>XEN_XX_GATA4_r2.bw<br>XEN_XX_GATA6_r1.bw<br>XEN_XX_GATA6_r2.bw<br>XEN_XX_H3K27ac_r1.bw<br>XEN_XX_H3K27ac_r2.bw<br>XEN_XX_H3K27me3_r1.bw<br>XEN_XX_H3K27me3_r2.bw<br>XEN_XX_H3K27me3_r3.bw<br>TS_XX_GATA2_r1.narrowPeak<br>TS_XX_GATA2_r2.narrowPeak<br>TS_XX_GATA3_r1.narrowPeak<br>TS_XX_GATA3_r2.narrowPeak<br>TS_XX_H3K27ac_r1.narrowPeak<br>TS_XX_H3K27ac_r2.narrowPeak<br>TS_XX_H3K27me3_r1.narrowPeak<br>TS_XX_H3K27me3_r2.narrowPeak<br>XEN_XX_GATA4_r1.narrowPeak<br>XEN_XX_GATA4_r2.narrowPeak<br>XEN_XX_GATA6_r1.narrowPeak<br>XEN_XX_GATA6_r2.narrowPeak<br>XEN_XX_H3K27ac_r1.narrowPeak<br>XEN_XX_H3K27ac_r2.narrowPeak<br>XEN_XX_H3K27me3_r1.narrowPeak<br>XEN_XX_H3K27me3_r2.narrowPeak<br>XEN_XX_H3K27me3_r3.narrowPeak<br>TS_XX_GATA2_r1_1.fastq.gz<br>TS_XX_GATA2_r2_1.fastq.gz<br>TS_XX_GATA3_r1_1.fastq.gz<br>TS_XX_GATA3_r2_1.fastq.gz<br>TS_XX_H3K27ac_r1_1.fastq.gz<br>TS_XX_H3K27ac_r2_1.fastq.gz<br>TS_XX_H3K27me3_r1_1.fastq.gz<br>TS_XX_H3K27me3_r2_1.fastq.gz<br>XEN_XX_GATA4_r1_1.fastq.gz<br>XEN_XX_GATA4_r2_1.fastq.gz<br>XEN_XX_GATA6_r1_1.fastq.gz<br>XEN_XX_GATA6_r2_1.fastq.gz<br>XEN_XX_H3K27ac_r1_1.fastq.gz<br>XEN_XX_H3K27ac_r2_1.fastq.gz<br>XEN_XX_H3K27me3_r1_1.fastq.gz<br>XEN_XX_H3K27me3_r2_1.fastq.gz<br>XEN_XX_H3K27me3_r3_1.fastq.gz<br>TS_XX_GATA2_r1_2.fastq.gz<br>TS_XX_GATA2_r2_2.fastq.gz<br>TS_XX_GATA3_r1_2.fastq.gz<br>TS_XX_GATA3_r2_2.fastq.gz<br>TS_XX_H3K27ac_r1_2.fastq.gz<br>TS_XX_H3K27ac_r2_2.fastq.gz<br>TS_XX_H3K27me3_r1_2.fastq.gz<br>TS_XX_H3K27me3_r2_2.fastq.gz<br>XEN_XX_GATA4_r1_2.fastq.gz<br>XEN_XX_GATA4_r2_2.fastq.gz<br>XEN_XX_GATA6_r1_2.fastq.gz

XEN_XX_GATA6_r2_2.fastq.gz
XEN_XX_H3K27ac_r1_2.fastq.gz
XEN_XX_H3K27ac_r2_2.fastq.gz
XEN_XX_H3K27me3_r1_2.fastq.gz
XEN_XX_H3K27me3_r2_2.fastq.gz
XEN_XX_H3K27me3_r3_2.fastq.gz

Genome browser session (e.g. UCSC): https://genome.ucsc.edu/s/Edda.schulz/Ravid_et_al_initialSubm

Methodology

Replicates: Two replicates were performed per condition, Pearson correlation coefficient between replicates was >0.95.

Sequencing depth: Samples were sequenced paired-end with 75bp on each side. 1-12 Mio total reads were sequenced per CUT&Tag library with >97% reads uniquely mapped. Detailed mapping statistics are provided in Suppl. Table 3.

Antibodies:
Gata2 Abcam ab109241
Gata3 BD Pharmingen 558686
Gata4 Santa Cruz sc-25310
Gata6 R&D Systems AF1700
H3K27me3 Active Motif 61017
H3K27ac Active Motif 39685

Peak calling parameters: Peaks for CUT&Tag and ChIP-seq were called using MACS2 (v2.1.2) with standard options [callpeak -f BAMPE/BAM -g mm -q 0.05] on individual replicates. For ChIP-seq, input samples were included for normalization using [-c]. Only peaks detected in all replicates were retained by merging replicates using bedtools (v2.29.2) with [intersect].

Data quality: Between 4300 and 56876 peaks were detected per library. To analyse specificity of the CUT&Tag experiments for GATA factors, we analysed motif enrichment in the identified peaks. This analysis is shown in Suppl. Fig. 5. A detailed summary of peak-calling statistics is provided in Suppl. Table 3.

Software: For CUT&Tag and ChIP-seq data, reads were trimmed for adapter sequences using Trim Galore (0.6.4) with options [--paired --nextera] or [--paired --illumina] (http://www.bioinformatics.babraham.ac.uk/projects/trim_galore/) prior to alignment. Read alignment was performed to the mm10 reference genome using bowtie2 (v2.3.5.1) with options [--local --very-sensitive-local --no-mixed --no-discordant --phred33 -I 10 -X 2000] (Langmead and Salzberg, 2012) for CUT&Tag/ChIP-seq or with STAR (v2.7.5a) with options [--outSAMattributes NH HI NM MD] (Dobin et al., 2013) for RNA-seq. Sequencing data was then filtered for properly mapped reads and sorted using samtools (Li et al., 2009) (v1.10) with options [view -f 2 -q 20] (CUT&Tag/ChIPseq) or [view -q 7 -f 3] (RNA-seq) and [sort]. For ChIP-seq/CUT&Tag, blacklisted regions for mm10 (ENCODE Project Consortium, 2012) were removed using bedtools (Quinlan and Hall, 2010) (v2.29.2) with options [intersect -v]. For ChIP-seq, reads were also deduplicated using Picard (v2.18.25) with options [MarkDuplicates VALIDATION_STRINGENCY=LENIENT REMOVE_DUPLICATES=TRUE] (http://broadinstitute.github.io/picard).

Flow Cytometry

Plots

Confirm that:

☒ The axis labels state the marker and fluorochrome used (e.g. CD4-FITC).

☒ The axis scales are clearly visible. Include numbers along axes only for bottom left plot of group (a 'group' is an analysis of identical markers).

☒ All plots are contour plots with outliers or pseudocolor plots.

☒ A numerical value for number of cells or percentage (with statistics) is provided.

Methodology

Sample preparation: A single cell suspension was created by trypsin treatment.

Instrument: Flow cytometry data was collected using a BD FACSAria II, BD FACSAria Fusion or BD FACS Celesta flow cytometer.

Software: Data was collected with the BD software and analyzed using RStudio with the flowCore (v1.52.1) and openCyto packages (v1.24.0).

Cell population abundance: Post-sort fractions were not analyzed.

Gating strategy

The sideward and forward scatter areas were used to discriminate cells from cells' debris, whereas the height and width of the sideward and forward scatter were used for doublet discrimination. For Flow-FISH, all cells that showed a fluorescence intensity above the 99th-percentile of the undifferentiated cell population control, which does not express Xist, were marked as Xist-positive. In the enhancer-reporter assay, the geometric mean of the GFP fluorescence intensity was calculated and background-corrected by subtracting the geometric mean of the TX-SP106 non-transduced control (GFP negative). An example of the gating strategy is shown in ED Fig. 3A.

☒ Tick this box to confirm that a figure exemplifying the gating strategy is provided in the Supplementary Information.
